# Associations of cumulative average dietary total antioxidant capacity and intake of antioxidants with metabolic syndrome risk in Korean adults aged 40 years and older: a prospective cohort study (KoGES_CAVAS)

**DOI:** 10.4178/epih.e2023067

**Published:** 2023-07-28

**Authors:** Ji-Sook Kong, Jiseon Lee, Youngjun Kim, Hye Won Woo, Min-Ho Shin, Sang Baek Koh, Hyeon Chang Kim, Yu-Mi Kim, Mi Kyung Kim

**Affiliations:** 1 Department of Preventive Medicine, Hanyang University College of Medicine, Seoul, Korea; 2 Institute for Health and Society, Hanyang University, Seoul, Korea; 3 Department of Public Health Sciences, Hanyang University, Seoul, Korea; 4 Department of Preventive Medicine, Chonnam National University Medical School, Gwangju, Korea; 5 Department of Preventive Medicine and Institute of Occupational Medicine, Yonsei Wonju College of Medicine, Wonju, Korea; 6 Department of Preventive Medicine, Yonsei University College of Medicine, Seoul, Korea

**Keywords:** Antioxidants, Flavonoids, Vitamins, Metabolic syndrome, Obesity, Cohort study

## Abstract

**OBJECTIVES:**

Limited and inconsistent prospective evidence exists regarding the relationship of dietary total antioxidant capacity (dTAC) and antioxidant intake with metabolic syndrome (MetS) risk. We evaluated the associations of the cumulative averages of dTAC and antioxidant intake (in 5 classes: retinol, vitamin C, vitamin E, carotenoids, and flavonoids, as well as 7 flavonoid subclasses) with the risk of MetS.

**METHODS:**

This study included 11,379 participants without MetS, drawn from the Korean Genome and Epidemiology Study_CArdioVascular disease Association Study (KoGES_CAVAS). The cumulative average consumption was calculated using repeated food frequency questionnaires. Incidence rate ratios were estimated using a modified Poisson regression model with a robust error estimator.

**RESULTS:**

The median follow-up period was 5.16 years, and 2,416 cases of MetS were recorded over 58,750 person-years. In men, significant inverse associations were observed in all 5 antioxidant classes, except for the highest quartile of dTAC. In women, dTAC and total flavonoids were not significantly associated with MetS; however, significant L-shaped associations were found for the remaining 4 antioxidant classes. Of the 7 flavonoid subclasses, only flavones in the highest quartile for men and flavan-3-ols in women lacked significant associations with MetS. The inverse associations were not sex-specific, but they were particularly pronounced among participants with a body mass index (BMI) of 23 kg/m^2^ or higher.

**CONCLUSIONS:**

The findings suggest that most antioxidant classes and flavonoid subclasses, unlike dTAC, exhibit a clear beneficial association with MetS in an L-shaped pattern in both men and women, particularly those with a high BMI.

## GRAPHICAL ABSTRACT

[Fig f2-epih-45-e2023067]

## INTRODUCTION

Metabolic syndrome (MetS) is a cluster of medical conditions that include abdominal obesity, insulin resistance, hypertension, and hyperlipidemia [1]. It is associated with an increased risk of mortality and morbidity, such as cardiovascular disease (CVD) and diabetes [2]. The prevalence of MetS fluctuates based on the definitions used, but it is estimated to affect 10% to 40% of the global population, equating to over 1 billion people worldwide [2,3]. Therefore, identifying modifiable risk factors for MetS could aid in reducing the global burden of the disease.

Oxidative stress may play a pathogenic role in the development and progression of MetS and its associated abnormalities [4]. Antioxidants can potentially counteract this oxidative damage by scavenging reactive free radicals in the body [5,6]. Extensive research has been conducted on the relationships of antioxidants or antioxidant-rich foods with various health outcomes [7]. However, these studies have been focused on individual antioxidants or specific foods, resulting in inconsistent findings. This inconsistency may stem in part from the varying types and amounts of antioxidants found in different foods, which can affect their overall antioxidant capacities [5,6]. To address these challenges, the concept of dietary total antioxidant capacity (dTAC) was introduced. This index represents the summed antioxidant capacity (AC) of all consumed foods [8]. Since its introduction, dTAC has been studied in relation to several health outcomes, including risk factors for CVD [9]. In Korea, research has also explored its cross-sectional association with dyslipidemia [10] and prospective association with diabetes [11]. However, limited evidence is available regarding the relationship between dTAC and MetS, with most studies being cross-sectional and primarily conducted in Western countries [9,12]. In addition to dTAC, the association between the antioxidants (5 antioxidant classes and 7 flavonoid subclasses) used to calculate dTAC [8] and MetS remains unclear [12]. A recent review suggested that, based on animal studies, the antioxidant quercetin may have beneficial effects on insulin resistance, inflammation, and blood pressure (BP) in obese individuals [13]. Our previous study on flavonoids and the risk of incident hypertension also revealed significant interactions of anthocyanins and proanthocyanidins with hypertension risk based on obesity status [14]. However, it remains unclear whether the associations of dTAC and antioxidant intake with MetS risk vary depending on obesity status.

Therefore, in this study, we aimed to assess the relationships of both dTAC and antioxidant intake (encompassing 5 classes: retinol, vitamin C, vitamin E, carotenoids, and flavonoids, as well as 7 flavonoid subclasses: flavonols, flavones, flavanones, flavan-3-ols, anthocyanins, isoflavones, and proanthocyanidins) with the risk of MetS among adults aged 40 years or older in a prospective cohort. Additionally, we attempted to identify any obesity-specific associations.

## MATERIALS AND METHODS

### Study design and population

The Korean Genome and Epidemiology Study_Cardiovascular Disease Association Study (KoGES_CAVAS) is a comprehensive investigation of cardiovascular health risk factors among Koreans, combining 3 distinct cohorts [15]. These cohorts include the MultiRural Communities Cohort (MRCohort), the Atherosclerosis Risk of Rural Areas in the Korean General Population (ARIRANG) cohort, and the Kangwha cohort. These cohorts have been included in the KoGES since 2005. Each cohort consists of community residents aged 40 years and older, recruited through multistage cluster sampling from 6 counties: Yangpyeong, Namwon, and Goryeong for the MRCohort; Wonju and Pyeongchang for ARIRANG; and Kangwha. Between 2005 and 2011, 19,546 participants with neither CVD nor cancer were enrolled in a baseline survey. Follow-up was conducted every 2-4 years from 2007 to 2017, with 78.2% of participants having more than 1 revisit.

We excluded participants with the following conditions at baseline: (1) those who already had prevalent MetS (n = 7,709) or lacked MetS identification information at baseline (n= 56); (2) those who left ≥ 10 items unanswered on the food frequency questionnaire (FFQ) or had an implausible energy intake (≤ 619 or ≥ 4,032 kcal/day in men and ≤ 509 or ≥ 3,918 kcal/day in women, representing cut-offs of the ≤ 0.5th or ≥ 99.5th percentiles of total energy intake) (n= 202); and (3) those with missing data for important covariates (n= 200), such as education level (n= 31), smoking status (n= 25), regular exercise (n= 123), drinking status (n= 17), and/or body mass index (BMI, kg/m^2^) (n= 12). The final analysis included 11,379 participants, with 4,422 men and 6,957 women.

### Ascertainment of metabolic syndrome

Five components of MetS were measured as follows. Waist circumference (WC) was determined at the midpoint between the bottom of the rib margin and the iliac crest. BP measurements were taken was measured twice from the right arm using a standard mercury sphygmomanometer in the MRCohort (Baumanometer; WA Baum Co., Inc., Copiague, NY, USA) and the ARIRIANG cohort (Baumanometer and CK-101, Spirit Medical Co., New Taipei City, Taiwan) and an automatic sphygmomanometer in the Kangwha cohort (Dinamap 1846 SX/P; Critikon, Tampa, FL, USA). These measurements were taken after the participants had been seated for at least 5 minutes to ensure stable readings. If a difference of more than 5 mmHg was observed between 2 consecutive systolic blood pressure (SBP) or diastolic blood pressure (DBP) readings, additional measurements were taken, and the average values were used for analysis. However, in the ARIRIANG cohort, more than half of the initial BP measurements were taken only once (56.2%) prior to joining KoGES_CAVAS, so these single readings were used. Blood samples were collected after a minimum of 8 hours of fasting. The serum concentrations of high-density lipoprotein cholesterol (HDL-C), triglycerides (TG), and fasting blood glucose (FBG) were then quantified using an ADVIA 1650 automated analyzer (Siemens, New York, NY, USA).

MetS was defined according to the National Cholesterol Education Program Adult Treatment Panel III (NCEP ATP III) criteria [16], but with a modified definition of abdominal obesity as per the Korean Society for the Study of Obesity [17]. To be categorized as having MetS, individuals had to meet at least 3 of the following 5 criteria at baseline: (1) a WC ≥ 90 cm for men and ≥ 85 cm for women; (2) elevated BP, specifically SBP ≥ 130 mmHg and/or DBP ≥ 85 mmHg, or the use of antihypertensive medication; (3) an elevated FBG level of ≥ 100 mg/dL or the use of medication for diabetes mellitus; (4) an elevated TG level, defined as ≥ 150 mg/dL; and (5) a reduced HDL-C level, defined as < 40 mg/dL for men and < 50 mg/dL for women.

Incident cases of MetS were identified using the same criteria as the baseline definition, namely the presence of at least 3 of the 5 risk factors at each follow-up visit. The earliest diagnosis of MetS was considered the incident event.

### Dietary assessment: total antioxidant capacity and nutrients

Food intake was assessed by highly trained interviewers using a validated FFQ comprising 106 food items. The validity of this FFQ was confirmed by de-attenuated correlation coefficients, which ranged from 0.23 for vitamin A (RE) to 0.64 for carbohydrates, with a median value of 0.39. The coefficients were 0.31 for beta-carotene and 0.34 for vitamin C [18]. Participants were asked to recall the frequency of consumption and average portion size of each food item they consumed over the past year. To aid comprehension and ensure study reliability, photographs of typical portion sizes were provided. The response options for each item included 9 frequency categories, from “never or rarely” to “3 times per day,” and 3 portion sizes. Daily nutrient intake was calculated using weighted frequencies per day and portion sizes per unit for each food item, based on the nutrient database found in the seventh edition of the Korean Food Composition Table [19].

To calculate dTAC, we constructed a database for antioxidants and total antioxidant capacity (TAC), incorporating 5 classes of antioxidants (retinol, vitamin C, vitamin E, carotenoids, and flavonoids) and 7 subclasses of flavonoids (flavonols, flavones, flavanones, flavan-3-ols, anthocyanins, isoflavones, and proanthocyanidins). We utilized food composition tables and databases provided by the government and authorized institutions. For vitamins and carotenoids, our primary source was the Food and Nutrient Database of the Korea Ministry of Food and Drug Safety [20], followed by the United States Department of Agriculture (USDA) Standard Reference (SR28, release 28) [21] and the Japanese Standard Tables of Food Composition (seventh revised edition) [22]. For flavonoids, we referenced 3 separate USDA databases—release 3.3 for flavonoids [23], release 2.1 for isoflavones [24], and release 2.1 for proanthocyanidins [25]—followed by the PhenolExplorer 3.6 database [26].

In total, 42 antioxidants were used: retinol, vitamin C, 4 forms of vitamin E (α-tocopherol, β-tocopherol, γ-tocopherol, and δ-tocopherol), 5 carotenoids (α-carotene, β-carotene, lycopene, β-cryptoxanthin, and lutein+zeaxanthin), and 31 flavonoids. Among the 31 flavonoids, 7 subclasses were included: 4 flavonols (quercetin, kaempferol, myricetin, and isorhamnetin), 2 flavones (luteolin and apigenin), 3 flavanones (hesperetin, naringenin, and eriodictyol), 10 flavan-3-ols (catechin, epicatechin, epigallocatechin, epicatechin 3-gallate, epigallocatechin 3-gallate, theaflavin, thearubigin, theaflavin 3-gallate, theaflavin 3´-gallate, and theaflavin 3,3´-digallate), 6 anthocyanins (cyanidin, delphinidin, malvidin, pelargonidin, peonidin, and petunidin), 4 isoflavones (daidzein, genistein, glycitein, and biochanin), and 2 proanthocyanidins (dimers and trimers). The antioxidant database covers approximately 60.9% of flavonoids, 92.2% of carotenoids, and over 99% of retinol, vitamin C, and vitamin E. The individual TAC of each food item (measured in mg vitamin C equivalent [VCE]/100 g) was calculated as the sum of the product of antioxidant content and antioxidant capacity of the individual antioxidants [8,27].

The dietary TAC and antioxidant intake for each participant were calculated using the TAC database, following the same method used to calculate nutrient intake. To better reflect long-term dietary consumption and reduce measurement errors [28], we calculated the cumulative averages of dTAC and antioxidant intake. This was achieved by averaging the intakes at baseline and during subsequent repeated examinations until each participant reached their endpoint or was censored. A maximum of 3 FFQs per person (average, 1.84), conducted at baseline and during follow-up visits, were utilized to calculate dTAC and antioxidants intake. All analyses employed the cumulative average of dTAC (mg VCE/day) and antioxidant intake (mg/day).

### Assessment of covariates

The interviewers and examiners of the 3 cohorts received training from trainers at the quality control center. To overcome the limitations inherent in a multicenter study, data collection strictly adhered to standard protocols for questionnaire surveys and examinations. Trained interviewers used a structured questionnaire to gather data on demographic characteristics such as age and education, as well as established risk factors for MetS, including regular exercise, smoking status, and current alcohol consumption. Daily alcohol consumption (in g/day) was calculated by multiplying the average frequency of consumption for 6 types of alcoholic beverages (soju, takju, beer, refined rice wine, wine, and whisky; in times/day) by the quantity consumed per occasion. This calculation considered the alcohol content (%) of each beverage and the specific gravity of alcohol (0.79). Height measurements were taken to the nearest 0.1 cm using a stadiometer, while weight was measured to the nearest 0.1 kg on a metric scale. These measurements were taken with participants dressed in light clothing and without shoes. BMI was then calculated as the ratio of weight (in kg) to the square of height (in m^2^). Participants were categorized into 2 groups based on obesity status: those with BMI < 23 kg/m^2^ and those with BMI ≥ 23 kg/m^2^.

### Statistical analysis

All analyses were conducted separately for men and women. The unadjusted baseline characteristics of the study population are presented as mean± standard deviation for quantitative variables and as numbers and percentages for categorical variables. Age-adjusted characteristics are presented as mean± standard error for quantitative variables and as percentages for categorical variables. We employed the general linear model to determine age-adjusted differences in baseline characteristics based on the quartiles (Q) of the cumulative averages of dTAC and antioxidants, using the lowest quartile (Q1) as the reference category. Tukey post-hoc tests were used to identify group differences at a significance level of p < 0.05. We used a modified Poisson regression model to estimate the incidence rate ratios (IRRs) and 95% confidence intervals (CIs) with a robust error estimator [29]. Three models were presented to adjust for potential confounders: (1) a model adjusted for age (in years); (2) multivariable model 1, which was additionally adjusted for potential confounders of MetS [30] such as higher education level (≥ 12 years of schooling, yes or no), regular exercise (≥3 times/wk and ≥30 min/session, yes or no), smoking status (current/past/never-smokers for men and current/non-smokers for women), current drinking status (yes or no), BMI (kg/m^2^), and total energy intake (kcal/day); and (3) multivariable model 2, which incorporated dietary factors previously reported to be associated with MetS [31], including the glycemic index (GI) and intake of calcium (mg/day), fiber (g/day), magnesium (mg/day), and sodium (mg/day) into multivariable model 1. A linear trend was obtained by treating the median value of each category of dTAC and antioxidant intake as a continuous value. We also examined the non-linear relationship of dTAC and individual antioxidant intake with the risk of MetS. Tests for non-linearity incorporated the Wald test to compare the deviance of the linear trend model to the deviance of the categorical model [32]. Restricted cubic spline analyses with 3 knots (at the 25th, 50th, and 75th percentiles) were conducted to explore possible non-linear associations. The models were adjusted for the same potential confounders as those in the final Poisson regression model. The results of these analyses are presented in Supplementary Materials 1 and 2. The association of each exposure variable with the MetS risk was analyzed by obesity status group. The potential interaction was assessed by adding the cross-product term of the exposure categories and gender or BMI group.

The final models confirmed the absence of multicollinearity in the variance inflation factor (VIF< 10), ensuring no multicollinearity among the independent variables [33]. We performed several sensitivity analyses to assess the robustness of our findings. First, we censored participants with a reported diagnosis of CVD or cancer (494 participants) between visits. This was done to minimize the potential influence of their treatments on MetS. Second, we conducted a sensitivity analysis that excluded any antioxidant supplement users of any antioxidant used when calculating the TAC (8.93%). Third, after selecting the major food sources that contributed 10% or more to the intake (%) or variation (r2) of dTAC and each antioxidant, we investigated the association of dTAC and antioxidants (classes and flavonoid subclasses) from those food sources with the risk of MetS incidence.

All statistical analyses were performed using SAS version 9.4 (SAS Institute Inc., Cary, NC, USA) and R version 4.0.0 (R Foundation for Statistical Computing, Vienna, Austria). A p-value of less than 0.05 was considered to indicate statistical significance.

### Ethics statement

The study was conducted in accordance with the principles of the Declaration of Helsinki, and the study protocol was approved by the Institutional Review Board of Hanyang University (HYU-2020-04-003-1). All participants provided written informed consent before participating in the study.

## RESULTS

A total of 2,416 *de novo* cases (930 men and 1,486 women) were recorded over 58,750 person-years (23,132 for men and 35,618 for women). The median follow-up period was 5.16 years (interquartile range, 1.97 to 7.86). The mean ages of the men and women were 59.7 years and 56.3 years, respectively (Supplemental Material 3). The cumulative average values of dTAC were 269 mg VCE/day for men and 316 mg VCE/day for women. Flavonoids made up 82% of the total dTAC, followed by vitamin C at 16.6%, carotenoids at 0.92%, and then vitamin E and retinol. Table 1 presents the age-adjusted characteristics of the study participants by dTAC quartile. Participants in the highest quartile (Q4) for dTAC tended to be relatively highly educated, never-smokers, and regular exercisers; additionally, they had a higher BMI and intake of total energy, calcium, fiber, magnesium, and sodium, along with a lower GI. Similar trends were also observed in Q4 of the antioxidant classes and flavonoid subclasses (data not shown).

Tables 2 and 3 present the associations of dTAC, the 5 antioxidant classes, and the 7 flavonoid subclasses with MetS incidence. After adjusting for covariates, including dietary factors, dietary TAC demonstrated an L-shaped association with the risk of MetS in men, although no significant association was observed in Q4. However, no significant association was found between dTAC and MetS in women. The intake levels of the following antioxidant classes were inversely associated with MetS incidence in both men and women, presented with IRR and 95% CI values for Q4 versus Q1: retinol (men: IRR, 0.65; 95% CI, 0.49 to 0.85; women: IRR, 0.72, 95% CI, 0.57 to 0.91), vitamin C (men: IRR, 0.56; 95% CI, 0.42 to 0.75; women: IRR, 0.69; 95% CI, 0.55 to 0.88), vitamin E (men: IRR, 0.76; 95% CI, 0.57 to 1.00; women: IRR, 0.75; 95% CI, 0.60 to 0.94), and carotenoids (men: IRR, 0.58; 95% CI, 0.46 to 0.74; women: IRR, 0.74; 95% CI, 0.60 to 0.91). Among the 5 antioxidant classes, total flavonoid intake was not significantly associated with the risk of MetS in women, unlike in men (IRR, 0.69; 95% CI, 0.54 to 0.88). However, an inverse trend was observed for the 7 subclasses of flavonoids, with the exception of flavones in men and flavan-3-ols in women (Table 3). Significant non-linear associations, possibly U-shaped or L-shaped, were found for all significant inverse associations (Supplemental Materials 1 and 2), although some significant linear relationships with the risk of MetS were also observed (p_linearity_< 0.001 for carotenoids in men, p_linearity_ < 0.001 for anthocyanin in men, p_linearity_< 0.001 for proanthocyanidins in men, and p_linearity_< 0.001 for flavonols in women based on the Bonferroni criteria of multiplicity [p< 0.05/26= 0.002]). For all associations between intake of antioxidants and MetS risk, no significant differences were observed between genders (all p_inter_≥ 0.05; data not shown).

In the sensitivity analyses, the inverse associations remained robust even after censoring CVD and cancers that developed during the follow-up period (494 participants) (Supplemental Material 4) and excluding supplement users of any antioxidants used to calculate dTAC (Supplemental Material 5). Dietary TAC and each antioxidant from most major food sources continued to show inverse associations with the risk of MetS incidence in both men and women. The exceptions were isoflavones from fermented soy products, which exhibited a null association, and vitamin C and flavones from *Baechu-kimchi*, which showed a positive association, but only in Q4 (Supplemental Material 6).

**Figure 1.**
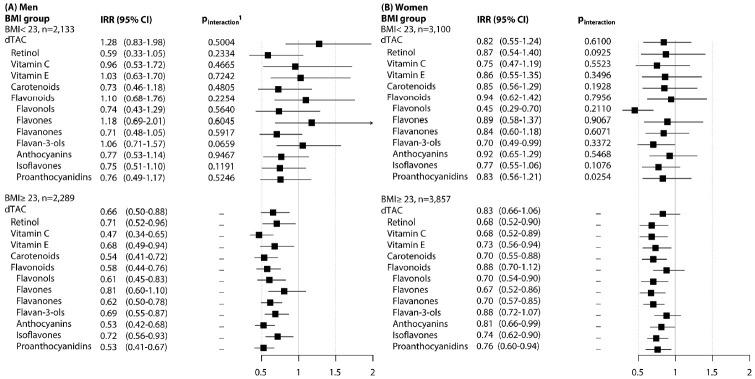
Incidence rate ratios (IRRs) of metabolic syndrome for low (quartile 1) versus high (quartile 4) intake levels by BMI group (A) men, and (B) women. dTAC, dietary total antioxidant capacity; BMI, body mass index. 1The p-interaction was tested by adding the cross-product term of the exposure categories and BMI group. The multivariable model was adjusted for age (years), higher education level (≥12 years), regular exercise (≥3 times/wk for ≥30 min/session), smoking status (current/past/never-smokers for men and current/non-smokers for women), drinking status (yes or no), BMI (kg/m^2^), total energy intake, glycemic index, calcium (mg/day), fiber (g/day), magnesium (mg/day), and sodium (mg/day) in men and women.

Figure 1 presents the associations of dTAC, antioxidant classes, and flavonoid subclasses with the risk of MetS, stratified by BMI group (overweight/obese vs. underweight/normal weight). The inverse associations persisted in participants who were overweight or obese compared to those of normal weight. However, a significant interaction was observed only for proanthocyanidins in women (p_inter_= 0.025), and this did not meet the significance threshold based on the Bonferroni criteria.

## DISCUSSION

We observed a weak association between the average dTAC and MetS in men, but no association in women. However, we found that the average intake of antioxidants, apart from total flavonoids in women, was inversely associated with the risk of MetS. Among the 7 subclasses of flavonoids, most were inversely associated with the risk of MetS in both men and women (Q4 vs. Q1), except for flavones in men and flavan-3-ols in women. These trends were characterized by non-linear, L-shaped relationships. We did not observe any gender-based interactions, and the beneficial associations for MetS were primarily seen in men and women who were relatively obese.

In this study, the average dTAC was 279.5 mg VCE/day; this is lower than the 384.7 mg VCE/day value reported for Korean adults aged 19 years and older, based on 24-hour recall data from the Korean National Health and Nutrition Examination Survey (KNHANES) [34]. However, relative to the dTAC values in the United States population (217.6 mg VCE/day for supplement non-users and 268.4 mg VCE/day for antioxidant supplement users) [35], the dTAC values observed in our study were high. The discrepancy in dTAC values, with the present finding being lower than the KNHANES but higher than the United States population measurement, could be attributed to several factors: (1) the use of different dietary assessment methods (FFQ in the present study versus 24-hour recall in the KNHANES), (2) the age group considered (≥ 40 years in the present study vs. ≥ 19 years in the KNHANES), and (3) variations in dietary pattern (a high intake of plant-based foods rich in flavonoids, including vegetables and legumes, in this study relative to the United States population) [36].

In the present study, the major food sources of dTAC and each antioxidant were as follows. Green tea (dTAC and total flavonoids), milk (retinol), and tomato/tomato juice (carotenoids) explained more than 50% of the variation in dietary intake. For the 7 flavonoids, orange/orange juice (flavanones), green tea (flavan-3-ols), grapes/grape juice (anthocyanins), and apples/apple juice (proanthocyanidins) contributed to over 50% of the variation in dietary intake (Supplemental Material 6). Depending on dietary patterns and food availability, the type and amount of antioxidants contributing to dTAC may vary across regions and countries [36]. However, the primary food sources of dTAC and antioxidants were sufficiently similar across studies, with fruits, vegetables, legumes, and beverages such as tea, wine, and juice being the main contributors [36].

In this study, we observed an L-shaped association between dTAC and the risk of MetS in men, although this was not significant for Q4. Moreover, as previously reported, no such association was present in women (p_inter_≥ 0.05) [12]. However, as recent crosssectional studies [37,38] and a prospective cohort study (the Tehran Lipid and Glucose Study) [39] have reported inverse associations of dTAC with MetS and its components, we conducted additional analyses on food sources to explore the potential for a weak or no association of dTAC in this study. First, we considered green tea, a primary food source of dTAC for both men and women. We found a significant L-shaped association for both genders. Second, we examined *Baechu-kimchi* consumption, as the vitamin C and flavones in this food showed positive associations with the risk of MetS in this study. When we adjusted for *Baechu-kimchi* consumption, a significant association was found even in Q4 of dTAC in men. After stratifying the *Baechu-kimchi* consumption by the median value, an inverse association was observed only in women with low *Baechu-kimchi* consumption (data not shown, p_inter_= 0.049). Notably, *Baechu-kimchi* is a major contributor of dietary salt in Korea, and the present study included a high proportion (70.8%) of postmenopausal women, who are known to be more sensitive to salt than premenopausal women [40]. Thus, it is possible that the protective effects of dTAC were insufficient to counteract the adverse effects of salt on cardiometabolic risks, particularly BP. Additionally, China and Poland, where studies have reported an inverse association between dTAC and MetS, exhibit lower salt intakes compared to Korea [41]. This suggests that the weak or absent association between dTAC and MetS in the present study may be partially due to the relatively high salt intake, which can lead to salt-dependent oxidative stress by eliminating the local nitric oxide activity and increasing microvascular levels of reactive oxygen species [42].

We observed an inverse association between total flavonoid consumption and MetS risk in men but not in women, with no significant gender-based specificity. Total flavonoids were the largest contributor to dTAC in the present study (mg VCE/d, 82.2% in men and 81.6% in women) and in representative Koreans from the KNHANES [34]. Total flavonoids were also found to be inversely associated with MetS risk in urban Polish cross-sectional [37] and Chinese prospective [43] studies. This association was observed for total flavonoids and flavanols (a combination of flavan-3-ol monomers and flavanol-derived compounds) in the Polish cross-sectional study [37] and for total flavonoid intake in the Chinese prospective study [43]. On the contrary, a cross-sectional Korean study showed no association of flavonoids with the prevalence risk of MetS [38]. Nevertheless, we found that intake of green tea, apples/apple juice, and grapes/grape juice, which were the major sources of flavonoids in this study, was inversely associated with the MetS risk, even in women (Supplemental Material 6). Like the association observed for dTAC in the stratification analysis of *Baechu-kimchi* consumption, total flavonoid intake was significantly and inversely associated with the MetS risk only in women with low *Baechu-kimchi* consumption (data not shown). Based on these findings, it is likely that flavonoids have a beneficial effect on reducing the risk of MetS.

Among flavonoid subclasses, flavones in men and flavan-3-ols in women were not significantly associated with MetS in Q4 in the present study. As we mentioned above, a major food source of flavones (*Baechu-kimchi*) was the only major source of antioxidants to exhibit a positive association with MetS. As for flavan3-ols, green tea, a major food source, appeared to have an inverse association with MetS; however, we could not explain why MetS was not associated with flavan-3-ol intake in women. That said, the inverse associations of 4 antioxidant vitamins (e.g., vitamin C, vitamin E, and carotenoids) with MetS risk were consistent with previous observational studies [44-46]. This could be explained by the direct effect of oxidative stress in the pathogenesis of MetS [4] and by the beneficial effect of antioxidant treatment on MetS components.

Most antioxidant classes and flavonoid subclasses displayed non-linear rather than linear associations, which was observed from a relatively low intake level (primarily Q2). Unfortunately, we were unable to establish a precise threshold for dietary antioxidant intake to prevent the onset of MetS; this was due to our method of collecting dietary data, which involved calculating relative intake from FFQ. However, the non-linearity was similar to that observed in a previous study on the association between antioxidant compounds, including flavonoids, and CVD risk [47]. This suggests that the trend may plateau, with no additional benefits beyond a certain intake level. Despite this, the dose-response relationship between dietary antioxidants and diseases, including MetS, remains ambiguous [4,7]. Further research is needed to establish reasonable standards or critical daily antioxidant intake doses. Furthermore, our findings indicate that these antioxidants are more effective in preventing MetS in overweight or obese individuals than in those who are underweight or of normal weight. Alongside growing evidence supporting the anti-obesity effects of antioxidants [48], these results suggest that the associations of dTAC, antioxidant classes, and flavonoid subclasses with MetS could be modified by baseline obesity status.

Several limitations should be considered when interpreting our results. First, our validated food-based FFQ was not specifically designed to assess antioxidants and dTAC, and it omitted a substantial portion of cooking oil and seasonings. Secondly, no universally accepted method is available to measure the antioxidant capacity of food, so values should be compared and interpreted with caution. Third, some measurement errors are unavoidable in dietary assessment using FFQ. The precise assessment of dietary intake and the large variability in antioxidant intake between different populations or nations present potential challenges in understanding the various functions of dTAC, antioxidant classes, and flavonoid subclasses [36]. Therefore, even though we used cumulative average intake to minimize random measurement errors, the accuracy of the antioxidant database must be enhanced. Fourth, our findings suggested that the inverse associations of antioxidant classes and flavonoid subclasses with MetS were more evident than those with dTAC. This implies that dTAC may not reflect the overall antioxidant capacity in the human body, which is influenced by a variety of endogenous and exogenous mechanisms [49]. Thus, although we used a validated algorithm to estimate dTAC [8], the algorithm should be refined by considering various factors that influence the antioxidant capacity of each food, such as cooking methods and added seasonings like salt. Finally, our analysis accounted for known confounding variables, such as total energy intake, glycemic index, calcium, fiber, magnesium, and sodium. However, other unmeasured confounding factors may exist. Furthermore, the current study did not include dyslipidemia medication in the definition of MetS, which could have led to the misclassification of participants taking dyslipidemia medication but with normal levels of TG or HDL-C. To address this limitation, we adjusted for dyslipidemia medications, and our findings remained robust. Despite these limitations, the present study exemplified a prospective design using repeated dietary assessment data and analyzed dTAC using a validated algorithm, subclass intake, and source foods.

In conclusion, the findings of this prospective study suggest that, with the exception of dTAC (Q4) and flavones (Q4) in men and dTAC, total flavonoids, and flavan-3-ol in women, 5 classes of antioxidants and 7 subclasses of flavonoids may help reduce the risk of developing MetS, especially in obese individuals. Moreover, after adjusting for or stratifying by the traditional Korean food, *Baechu-kimchi*, we noted a more pronounced inverse relationship of dTAC and total flavonoids with the risk of MetS. Therefore, future research should employ innovative methods to account for the complexity of food to reflect dietary cultures, such as cooking methods and the interplay between food and nutrient intake.

## Figures and Tables

**Figure f2-epih-45-e2023067:**
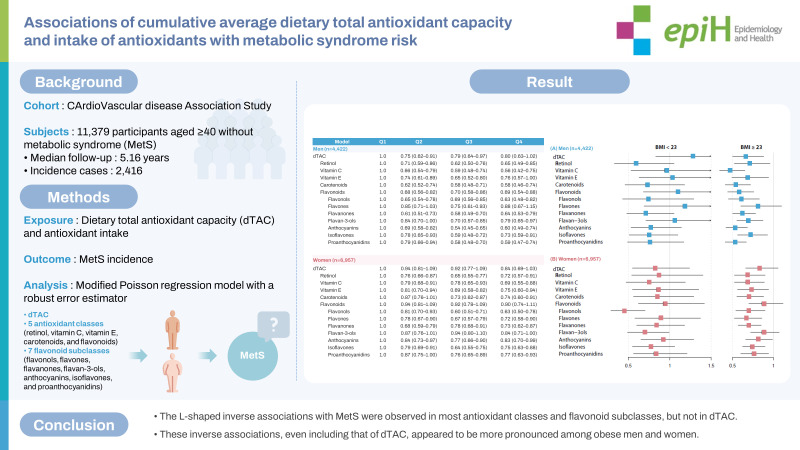


**Table 1.** Age-adjusted characteristics of study participants by quartile of dTAC

Characteristics		Men (n=4,422)				p diff^1^	p trend^2^	Women (n=6,957)				p diff^1^	p trend^2^
		Q1	Q2	Q3	Q4			Q1	Q2	Q3	Q4		
Total (n)		1,105	1,106	1,106	1,105			1,739	1,739	1,740	1,739		
Median (Min-Max) of dTAC (mg VCE/day)		76.5 (6.1-109.4)	142.4 (109.4-183.7)	232.3 (183.0-325.8)	505.8 (325.0-4,537.5)			84.6 (2.0-123.5)	162.8 (124.5-210.4)	276.1 (210.4-381.2)	607.2 (381.2-5,056.3)		
Age (yr)		62.6±10.0^a^	60.7±9.3^b^	59.1±9.4^c^	56.3±9.1^d^	<0.001	<0.001	61.2±10.4^a^	57.4±9.5^b^	54.3±8.7^c^	52.1±8.1^d^	<0.001	<0.001
Higher education^3^		25.4	31.5	38.7	46.2	<0.001	<0.001	20.5	23.6	30.3	36.6	<0.001	<0.001
Regular exercise^4^		13.0	17.2	23.2	29.9	<0.001	<0.001	14.3	19.4	26.2	33.1	<0.001	<0.001
Smoking status													
	Never-smoker	26.4	26.3	31.2	32.5	0.001	<0.001	94.5	97.2	97.2	95.3	<0.001	0.533
	Past smoker	31.5	33.7	32.8	38.4	0.005	0.001	1.2	0.8	0.9	1.6	0.092	0.079
	Current smoker	42.1	40.1	36.1	29.2	<0.001	<0.001	4.3	2.0	1.9	3.1	<0.001	0.695
Current drinker		64.0	64.5	62.3	67.3	0.112	0.084	28.4	29.4	29.4	32.7	0.047	0.006
Alcohol consumption (g/day)		23.1±1.3	20.7±1.3	21.2±1.3	22.0±1.3	0.575	0.905	1.8±0.2	1.8±0.2	1.8±0.2	2.4±0.2	0.106	0.021
Body mass index (kg/m^2^)		22.9±0.1^a^	23.1±0.1^a^	23.1±0.1^a^	23.5±0.1^b^	<0.001	<0.001	23.3±0.1^a^	23.5±0.1^a^	23.6±0.1^a^,^b^	23.8±0.1^b^	<0.001	<0.001
Dietary factors^5^													
	Total energy intake (kcal/day)	1,398.2±11.5^a^	1,589.3±11.4^b^	1,801.3±11.4^c^	1,902.1±11.5^d^	<0.001	<0.001	1,242.2±9.2^a^	1,448.2±8.9^b^	1,591.5±8.9^c^	1,745.3±9.1^d^	<0.001	<0.001
	Glycemic index	58.5±0.1^a^	57.5±0.1^b^	56.9±0.1^c^	56.3±0.1^d^	<0.001	<0.001	58.8±0.1^a^	57.7±0.1^b^	56.6±0.1^c^	55.5±0.1^d^	<0.001	<0.001
	Calcium (mg/day)	274.6±3.9^a^	333.4±3.9^b^	369.5±3.9^c^	405.7±3.9^d^	<0.001	<0.001	274.1±3.5^a^	328.5±3.4^b^	370.0±3.4^c^	421.0±3.4^d^	<0.001	<0.001
	Fiber (g/day)	12.8±0.1^a^	15.3±0.1^b^	16.4±0.1^c^	17.6±0.1^d^	<0.001	<0.001	11.8±0.1^a^	14.2±0.1^b^	15.9±0.1^c^	17.2±0.1^d^	<0.001	<0.001
	Magnesium (mg/day)	92.9±0.5^a^	93.8±0.5^a^	95.6±0.5^b^	98.4±0.6^c^	<0.001	<0.001	83.6±0.4^a^	84.3±0.4^a^	86.9±0.4^b^	92.1±0.4^c^	<0.001	<0.001
	Sodium (mg/day)	2,487.2±41.9^a^	2,885.8±41.4^b^	2,975.8±41.4^a^	3,128.1±42.0^c^	<0.001	<0.001	2,033.3±30.7^a^	2,362.8±29.7^b^	2,488.1±29.8^c^	2,648.0±30.4^d^	<0.001	<0.001

Values are presented as mean±standard error for continuous variables and % for categorical variables; Mean values with different superscripts (a, b, c) within a row exhibited significant differences among the exposure groups on the Tukey multiple comparison test. dTAC, dietary total antioxidant capacity; VCE, vitamin C equivalent; Q, quartile; Min, minimum; Max, maximum.

1 Using the general linear model (Tukey multiple comparison).

2 Linear trends were obtained by treating the median value of each group as a continuous variable.

3 Higher education level (≥12 years of schooling).

4 Regular exercise (≥3 times/wk and ≥30 min/session).

5 Nutrient intakes were adjusted for total energy intake using the residual method.

**Table 2.** Incidence rate ratios and 95% confidence intervals of MetS by quartile of dTAC and intake of 5 antioxidant classes (n=11,379)

Model^1^		Men (n=4,422)				p_linearity_^2^	p_non-linearity_^3^	Women (n=6,957)				p_linearity_^2^	p_non-linearity_^3^
		Q1	Q2	Q3	Q4			Q1	Q2	Q3	Q4		
dTAC (mg VCE/day)													
	Median (Min-Max)	76.5 (6.1-109.4)	142.4 (109.4-182.7)	232.3 (183.0-324.8)	504.8 (325.0-4,537.5)			84.6 (2.0-123.5)	161.8 (123.5-210.4)	276.1 (210.4-381.2)	607.2 (381.2-5,056.3)		
	Cases/PY	242/5,348	216/5,996	228/5,977	244/5,811			387/8,149	387/9,068	372/9,280	340/9,122		
	Age-adjusted model	1.00 (reference)	0.80 (0.67, 0.95)	0.84 (0.71, 1.00)	0.93 (0.77,1.11)	0.866	0.023	1.00 (reference)	1.02 (0.89, 1.17)	1.06 (0.92, 1.23)	1.07 (0.92, 1.24)	0.405	0.845
	Multivariable model 1	1.00 (reference)	0.77 (0.64, 0.92)	0.81 (0.67, 0.98)	0.82 (0.67, 1.01)	0.424	0.017	1.00 (reference)	0.98 (0.85, 1.13)	0.98 (0.84, 1.14)	0.91 (0.77, 1.08)	0.261	0.960
	Multivariable model 2	1.00 (reference)	0.75 (0.62, 0.91)	0.79 (0.64, 0.97)	0.80 (0.63, 1.02)	0.583	0.012	1.00 (reference)	0.94 (0.81, 1.09)	0.92 (0.77, 1.09)	0.84 (0.69, 1.03)	0.110	0.872
Retinol (mg/day)													
	Median (Min-Max)	0.01 (0.00-0.02)	0.03 (0.02-0.05)	0.07 (0.05-0.09)	0.14 (0.09-0.74)			0.01 (0.00-0.02)	0.03 (0.02-0.05)	0.07 (0.05-0.10)	0.14 (0.10-0.87)		
	Cases/PY	269/5,305	220/5,841	209/6,208	232/5,778			457/7,924	360/9,187	325/9,802	344/8,705		
	Age-adjusted model	1.00 (reference)	0.75 (0.67, 0.83)	0.66 (0.59, 0.74)	0.80 (0.72, 0.90)	0.062	<0.001	1.00 (reference)	0.77 (0.67, 0.88)	0.69 (0.60, 0.79)	0.84 (0.73, 0.98)	0.119	<0.001
	Multivariable model 1	1.00 (reference)	0.74 (0.66, 0.83)	0.65 (0.58, 0.73)	0.74 (0.65, 0.85)	0.014	0.001	1.00 (reference)	0.76 (0.66, 0.87)	0.66 (0.57, 0.77)	0.77 (0.65, 0.91)	0.016	<0.001
	Multivariable model 2	1.00 (reference)	0.71 (0.59, 0.86)	0.62 (0.50, 0.76)	0.65 (0.49, 0.85)	0.021	<0.001	1.00 (reference)	0.76 (0.66, 0.87)	0.65 (0.55, 0.77)	0.72 (0.57, 0.91)	0.029	<0.001
Vitamin C (mg/day)													
	Median (Min-Max)	18.6 (1.4-25.0)	30.8 (25.0-37.1)	44.7 (37.1-54.8)	72.0 (54.9-432.7)			20.5 (0.5-28.6)	36.6 (28.6-44.7)	54.3 (44.7-66.7)	88.9 (66.7-391.2)		
	Cases/PY	252/5,334	221/5,994	229/6,111	228/5,693			405/8,090	373/9,291	357/9,192	351/9,045		
	Age-adjusted model	1.00 (reference)	0.78 (0.65, 0.93)	0.79 (0.66, 0.94)	0.84 (0.71, 1.01)	0.215	0.017	1.00 (reference)	0.90 (0.78, 1.03)	0.97 (0.83, 1.12)	1.01 (0.87, 1.17)	0.550	0.227
	Multivariable model 1	1.00 (reference)	0.72 (0.60, 0.86)	0.69 (0.57, 0.83)	0.70 (0.57, 0.87)	0.011	0.002	1.00 (reference)	0.85 (0.74, 0.98)	0.89 (0.76, 1.04)	0.87 (0.73, 1.04)	0.295	0.125
	Multivariable model 2	1.00 (reference)	0.66 (0.54, 0.79)	0.59 (0.48, 0.74)	0.56 (0.42, 0.75)	0.002	<0.001	1.00 (reference)	0.79 (0.68, 0.91)	0.78 (0.65, 0.93)	0.69 (0.55, 0.88)	0.011	0.057
Vitamin E (mg/day)													
	Median (Min-Max)	2.0 (0.0- 2.8)	3.5 (2.8-4.3)	5.2 (4.3-6.3)	8.2 (6.3-27.6)			1.6 (0.0-2.4)	3.1 (2.4-3.9)	4.8 (3.9-6.0)	7.9 (6.0-36.9)		
	Cases/PY	250/5,286	225/6,041	214/6,205	241/5,600			431/8,164	376/9,263	326/9,418	353/8,773		
	Age-adjusted model	1.00 (reference)	0.79 (0.66, 0.93)	0.73 (0.61, 0.87)	0.91 (0.76, 1.08)	0.514	0.001	1.00 (reference)	0.88 (0.77, 1.01)	0.79 (0.68, 0.92)	0.97 (0.84, 1.13)	0.830	0.002
	Multivariable model 1	1.00 (reference)	0.74 (0.62, 0.89)	0.65 (0.53, 0.79)	0.76 (0.60, 0.97)	0.087	<0.001	1.00 (reference)	0.84 (0.73, 0.97)	0.74 (0.63, 0.86)	0.86 (0.71, 1.03)	0.121	0.001
	Multivariable model 2	1.00 (reference)	0.74 (0.61, 0.89)	0.65 (0.52, 0.80)	0.76 (0.57, 1.00)	0.183	<0.001	1.00 (reference)	0.81 (0.70, 0.94)	0.69 (0.58, 0.82)	0.75 (0.60, 0.94)	0.026	0.001
Carotenoids (mg/day)													
	Median (Min-Max)	2.1 (0.0-3.1)	4.0 (3.1-5.0)	6.2 (5.0-8.0)	11.0 (8.0-119.3)			2.3 (0.0-3.5)	4.7 (3.5-5.9)	7.3 (5.9-9.3)	12.8 (9.3-120.3)		
	Cases/PY	266/5,286	210/5,979	223/6,115	231/5,752			396/8,009	390/9,140	338/9,376	362/9,093		
	Age-adjusted model	1.00 (reference)	0.70 (0.59, 0.83)	0.72 (0.61, 0.86)	0.80 (0.67, 0.95)	0.127	<0.001	1.00 (reference)	0.94 (0.82, 1.08)	0.86 (0.74, 1.00)	0.99 (0.85, 1.15)	0.978	0.073
	Multivariable model 1	1.00 (reference)	0.65 (0.55, 0.78)	0.63 (0.53, 0.76)	0.66 (0.54, 0.81)	0.003	<0.001	1.00 (reference)	0.92 (0.80, 1.06)	0.81 (0.69, 0.94)	0.87 (0.74, 1.03)	0.125	0.059
	Multivariable model 2	1.00 (reference)	0.62 (0.52, 0.74)	0.58 (0.48, 0.71)	0.58 (0.46, 0.74)	0.001	<0.001	1.00 (reference)	0.87 (0.76, 1.01)	0.73 (0.62, 0.87)	0.74 (0.60, 0.91)	0.006	0.040
Flavonoids (mg/day)													
	Median (Min-Max)	44.8 (2.2-66.0)	87.4 (66.0-111.9)	144.9 (112.1-193.5)	293.7 (193.6-1,982.4)			50.5 (0.7-75.6)	101.8 (75.6-134.6)	177.1 (134.7-239.8)	350.6 (239.8-2,484.9)		
	Cases/PY	255/5,264	211/6,012	230/6,039	234/5,817			392/8,029	380/9,202	373/9,444	341/8,943		
	Age-adjusted model	1.00 (reference)	0.72 (0.61, 0.86)	0.78 (0.66, 0.93)	0.83 (0.69, 0.99)	0.351	0.001	1.00 (reference)	0.98 (0.85, 1.12)	1.02 (0.88, 1.18)	1.06 (0.91, 1.24)	<0.001	0.869
	Multivariable model 1	1.00 (reference)	0.70 (0.58, 0.83)	0.72 (0.60, 0.87)	0.72 (0.58, 0.88)	0.183	0.001	1.00 (reference)	0.96 (0.83, 1.11)	0.95 (0.81, 1.11)	0.94 (0.79, 1.12)	0.121	0.868
	Multivariable model 2	1.00 (reference)	0.68 (0.56, 0.82)	0.70 (0.58, 0.86)	0.69 (0.54, 0.88)	0.118	<0.001	1.00 (reference)	0.94 (0.81, 1.09)	0.92 (0.78, 1.09)	0.90 (0.74, 1.11)	0.435	0.764

MetS, metabolic syndrome; VCE, vitamin C equivalents; dTAC, dietary total antioxidant capacity; Q, quartile; Min, minimum; Max, maximum; PY, person-year.

1 Multivariable model 1 was adjusted for age (years), higher education level (≥12 years of schooling), regular exercise (≥3 times/wk and ≥30 min/session), smoking status (current/past/never-smokers for men and current/non-smokers for women), drinking status (yes or no), body mass index (kg/m^2^), and total energy intake (kcal/day) in men and women.

Multivariable model 2 was adjusted for glycemic index, calcium (mg/day), fiber (g/day), magnesium (mg/day), sodium (mg/day), and all covariates in multivariable model 1.

2 Linear trends were obtained by treating the median value of each group as a continuous variable.

3 Non-linear trends were obtained by comparing the deviance difference between the linear trend model with 1 degree of freedom and the ℓ ordered categorical model with ℓ−1 degrees of freedom.

**Table 3.** Incidence rate ratios and 95% confidence intervals of MetS by quartile of intake of 7 flavonoid subclasses (n=11,379)

Model^1^		Men (n=4,422)				p_linearity_^2^	p_non-linearity_^3^	Women (n=6,957)				p_linearity_^2^	p_non-linearity_^3^
		Q1	Q2	Q3	Q4			Q1	Q2	Q3	Q4		
Flavonols (mg/day)													
	Median (Min- Max)	6.8 (0.2-9.4)	11.9 (9.4-14.5)	17.7 (14.6- 22.1)	29.3 (22.1-245.7)			6.3 (0.0-9.0)	11.5 (9.0-14.5)	17.7 (14.5-22.8)	31.8 (22.8-219.2)		
	Cases/PY	240/5,296	202/6,131	247/6,139	241/5,566			415/8,042	392/9,258	321/9,570	358/8,748		
	Age-adjusted model	1.00 (reference)	0.73 (0.61, 0.87)	0.89 (0.75, 1.06)	0.95 (0.80, 1.14)	0.520	0.001	1.00 (reference)	0.93 (0.81, 1.06)	0.79 (0.68, 0.91)	1.01 (0.87, 1.17)	0.720	0.001
	Multivariable model 1	1.00 (reference)	0.69 (0.57, 0.82)	0.78 (0.64, 0.93)	0.78 (0.64, 0.95)	0.183	0.001	1.00 (reference)	0.88 (0.76, 1.01)	0.71 (0.61, 0.82)	0.85 (0.73, 1.01)	0.121	<0.001
	Multivariable model 2	1.00 (reference)	0.65 (0.54, 0.78)	0.69 (0.56, 0.85)	0.63 (0.48, 0.82)	0.019	<0.001	1.00 (reference)	0.81 (0.70, 0.93)	0.60 (0.51, 0.71)	0.63 (0.50, 0.78)	<0.001	<0.001
Flavones (mg/day)													
	Median (Min-Max)	0.7 (0.0-0.9)	1.2 (0.9-1.4)	1.7 (1.4-2.1)	2.7 (2.1-12.6)			0.7 (0.0-1.0)	1.3 (1.0-1.6)	1.9 (1.6-2.4)	3.3 (2.4-14.9)		
	Cases/PY	221/5,424	236/6,018	221/6,148	252/5,542			419/8,125	367/9,155	337/9,526	363/8,812		
	Age-adjusted model	1.00 (reference)	0.96 (0.81, 1.15)	0.88 (0.74, 1.06)	1.12 (0.94, 1.34)	0.174	0.079	1.00 (reference)	0.89 (0.77, 1.02)	0.84 (0.73, 0.98)	1.05 (0.90, 1.22)	0.316	0.006
	Multivariable model 1	1.00 (reference)	0.88 (0.74, 1.06)	0.79 (0.65, 0.96)	0.95 (0.77, 1.17)	0.818	0.030	1.00 (reference)	0.83 (0.72, 0.96)	0.75 (0.64, 0.87)	0.88 (0.74, 1.04)	0.300	0.001
	Multivariable model 2	1.00 (reference)	0.85 (0.71, 1.03)	0.75 (0.61, 0.93)	0.88 (0.67, 1.15)	0.588	0.014	1.00 (reference)	0.78 (0.67, 0.90)	0.67 (0.57, 0.79)	0.72 (0.58, 0.90)	0.018	<0.001
Flavanones (mg/day)													
	Median (Min-Max)	0.7 (0.0-1.7)	2.8 (1.7-4.2)	5.9 (4.2, 8.5)	12.9 (8.5-137.6)			1.2 (0.0-2.8)	4.5 (2.8-6.5)	9.1 (6.6-12.8)	19.5 (12.8-192.4)		
	Cases/PY	279/4,884	215/6,123	213/6,277	223/5,848			434/7,537	347/9,474	372/9,389	333/9,218		
	Age-adjusted model	1.00 (reference)	0.61 (0.52, 0.73)	0.59 (0.50, 0.70)	0.66 (0.56, 0.79)	0.003	<0.001	1.00 (reference)	0.70 (0.61, 0.81)	0.82 (0.71, 0.94)	0.80 (0.69, 0.93)	0.134	<0.001
	Multivariable model 1	1.00 (reference)	0.61 (0.51, 0.72)	0.57 (0.48, 0.68)	0.62 (0.52, 0.75)	0.001	<0.001	1.00 (reference)	0.69 (0.60, 0.79)	0.79 (0.68, 0.91)	0.75 (0.64, 0.88)	0.035	<0.001
	Multivariable model 2	1.00 (reference)	0.61 (0.51, 0.73)	0.58 (0.49, 0.70)	0.64 (0.53, 0.79)	0.004	<0.001	1.00 (reference)	0.68 (0.59, 0.79)	0.78 (0.68, 0.91)	0.73 (0.62, 0.87)	0.033	<0.001
Flavan-3-ols (mg/day)													
	Median (Min-Max)	2.9 (0.0-5.8)	9.6 (5.8-16.1)	28.0 (16.1, 47.9)	127.7 (48.0-1,755.8)			3.4 (0.0-7.1)	11.8 (7.1-19.0)	33.9 (19.0-57.6)	139.2 (57.7-1,808.2)		
	Cases/PY	247/5,179	230/5,951	207/5,935	246/6,066			402/7,849	367/9,198	373/9,025	344/9,546		
	Age-adjusted model	1.00 (reference)	0.81 (0.68, 0.96)	0.73 (0.61, 0.87)	0.84 (0.71, 1.00)	0.682	0.002	1.00 (reference)	0.88 (0.77, 1.02)	1.00 (0.87, 1.16)	0.93 (0.80, 1.08)	0.748	0.130
	Multivariable model 1	1.00 (reference)	0.82 (0.69, 0.98)	0.69 (0.57, 0.83)	0.79 (0.65, 0.95)	0.353	0.001	1.00 (reference)	0.88 (0.76, 1.02)	0.95 (0.82, 1.10)	0.86 (0.73, 1.00)	0.145	0.214
	Multivariable model 2	1.00 (reference)	0.84 (0.70, 1.00)	0.70 (0.57, 0.85)	0.79 (0.65, 0.97)	0.409	0.002	1.00 (reference)	0.87 (0.76, 1.01)	0.94 (0.80, 1.10)	0.84 (0.71, 1.00)	0.130	0.190
Anthocyanins (mg/day)													
	Median (Min-Max)	0.8 (0.0-1.7)	2.9 (1.7-4.2)	5.9 (4.2-8.3)	13.7 (8.3-149.9)			1.2 (0.0-2.5)	4.0 (2.5-5.9)	8.2 (5.9-12.3)	19.4 (12.3-224.7)		
	Cases/PY	274/4,949	233/5,989	200/6,146	223/6,047			418/7,784	373/9,331	348/9,519	347/8,984		
	Age-adjusted model	1.00 (reference)	0.70 (0.59, 0.83)	0.58 (0.49, 0.70)	0.66 (0.56, 0.79)	0.001	<0.001	1.00 (reference)	0.83 (0.72, 0.96)	0.82 (0.71, 0.95)	0.90 (0.77, 1.04)	0.557	0.010
	Multivariable model 1	1.00 (reference)	0.69 (0.58, 0.82)	0.54 (0.44, 0.65)	0.60 (0.49, 0.72)	<0.001	<0.001	1.00 (reference)	0.85 (0.74, 0.98)	0.78 (0.67, 0.91)	0.85 (0.73, 1.00)	0.215	0.009
	Multivariable model 2	1.00 (reference)	0.69 (0.58, 0.82)	0.54 (0.45, 0.65)	0.60 (0.49, 0.74)	<0.001	<0.001	1.00 (reference)	0.84 (0.73, 0.97)	0.77 (0.66, 0.90)	0.83 (0.70, 0.99)	0.194	0.007
Isoflavones (mg/day)													
	Median (Min-Max)	5.8 (0.2-8.7)	11.7 (8.7-14.9)	19.5 (14.9-25.9)	37.8 (25.9-157.1)			5.8 (0.0-8.5)	11.3 (8.5-14.5)	19.3 (14.5-25.1)	35.7 (25.1-190.8)		
	Cases/PY	247/5,025	244/5,961	205/6,437	234/5,709			415/7,824	368/9,041	319/9,671	384/9,082		
	Age-adjusted model	1.00 (reference)	0.83 (0.70, 0.99)	0.65 (0.54, 0.77)	0.83 (0.70, 0.99)	0.119	<0.001	1.00 (reference)	0.83 (0.72, 0.95)	0.69 (0.60, 0.80)	0.88 (0.77, 1.01)	0.239	<0.001
	Multivariable model 1	1.00 (reference)	0.78 (0.66, 0.94)	0.60 (0.49, 0.72)	0.75 (0.62, 0.91)	0.032	<0.001	1.00 (reference)	0.81 (0.70, 0.93)	0.67 (0.58, 0.78)	0.81 (0.70, 0.95)	0.055	<0.001
	Multivariable model 2	1.00 (reference)	0.78 (0.65, 0.93)	0.59 (0.48, 0.72)	0.73 (0.59, 0.91)	0.044	<0.001	1.00 (reference)	0.79 (0.69, 0.91)	0.64 (0.55, 0.75)	0.75 (0.63, 0.88)	0.010	<0.001
Proanthocyanidins (mg/day)													
	Median (Min-Max)	10.7 (0.0-18.4)	25.7 (18.4-34.2)	45.8 (34.3-61.7)	89.6 (61.7-1,138.7)			14.7 (0.0-24.0)	34.0 (24.1-45.3)	61.6 (45.3-85.4)	126.5 (85.4-836.5)		
	Cases/PY	253/5,017	245/5,873	215/6,203	217/6,038			411/7,855	392/9,090	346/9,537	337/9,136		
	Age-adjusted model	1.00 (reference)	0.82 (0.70, 0.98)	0.68 (0.57, 0.82)	0.71 (0.59, 0.85)	<0.001	0.014	1.00 (reference)	0.91 (0.80, 1.05)	0.83 (0.71, 0.95)	0.89 (0.77, 1.04)	0.207	0.075
	Multivariable model 1	1.00 (reference)	0.80 (0.67, 0.95)	0.59 (0.49, 0.71)	0.60 (0.49, 0.73)	<0.001	0.001	1.00 (reference)	0.87 (0.76, 1.00)	0.78 (0.67, 0.91)	0.81 (0.68, 0.95)	0.034	0.039
	Multivariable model 2	1.00 (reference)	0.79 (0.66, 0.94)	0.58 (0.48, 0.70)	0.59 (0.47, 0.74)	<0.001	<0.001	1.00 (reference)	0.87 (0.75, 1.00)	0.76 (0.65, 0.89)	0.77 (0.63, 0.93)	0.023	0.036

MetS, metabolic syndrome; Q, quartile; Min, minimum; Max, maximum; PY, person-year.

1 Multivariable model 1 was adjusted for age (years), higher education level (≥12 years of schooling), regular exercise (≥3 times/wk and ≥30 min/session), smoking status (current/past/never-smokers for men and current/non-smokers for women), drinking status (yes or no), body mass index (kg/m^2^), and total energy intake (kcal/day) in men and women.

Multivariable model 2 was adjusted for glycemic index, calcium (mg/day), fiber (g/day), magnesium (mg/day), sodium (mg/day), and all covariates in multivariable model 1.

2 Linear trends were obtained by treating the median value of each group as a continuous variable.

3 Non-linear trends were obtained by comparing the deviance difference between the linear trend model with 1 degree of freedom and the ℓ ordered categorical model with ℓ−1 degrees of freedom.

## SUPPLEMENTARY MATERIALS

Supplementary Material 1. 1.1. Nonlinear dose-response association of dTAC and antioxidants with incident MetS in men: a restricted cubic spline analysis with three knots (located at the 25th, 50th, and 75th percentiles) 1.2. Nonlinear dose-response association of dTAC and antioxidant classes with incident MetS in women: a restricted cubic spline analysis with three knots (located at the 25th, 50th, and 75th percentiles)

Supplementary Material 2 2.1. Nonlinear dose-response association of total flavonoids and flavonoid subclasses with incident MetS in men: a restricted cubic spline analysis with three knots (located at the 25th, 50th, and 75th percentiles) 2.2. Nonlinear dose-response association of total flavonoids and flavonoid subclasses with incident MetS in women: a restricted cubic spline analysis with three knots (located at the 25th, 50th, and 75th percentiles)

Supplementary Material 3 Baseline characteristics of the study participants (n=11,379)

Supplementary Material 4 Incidence rate ratios (IRRs) and 95% confidence intervals (CIs) of MetS by the quartiles of dTAC and antioxidant classes after censoring CVD and cancer (n=11,379)

Supplementary Material 5 Incidence rate ratios (IRRs) and 95% confidence intervals (CIs) of MetS by quartiles of dTAC and antioxidant classes among only non-users of antioxidant component supplement (n=8,786)

Supplementary Material 6 Incidence rate ratios (IRRs) and 95% confidence intervals (CIs) of MetS by quartiles of dTAC and antioxidant classes of their major food sources (n=11,379)
