# Supplementary material for: Associations of cumulative average dietary total antioxidant capacity and intake of antioxidants with metabolic syndrome risk in Korean adults aged 40 years and older: a prospective cohort study (KoGES_CAVAS)
Source: Epidemiol Health. 2023 Jul 28;45:e2023067. doi: 10.4178/epih.e2023067 (PMC10667584; doi:10.4178/epih.e2023067)
Supplement: Supplementary Material 2 — 2.1. Nonlinear dose-response association of total flavonoids and flavonoid subclasses with incident MetS in men: a restricted cubic spline analysis with three knots (located at the 25th, 50th, and 75th percentiles) 2.2. Nonlinear dose-response association of total flavonoids and flavonoid subclasses with incident MetS in women: a restricted cubic spline analysis with three knots (located at the 25th, 50th, and 75th percentiles) [file epih-45-e2023067-Supplementary-2.docx]

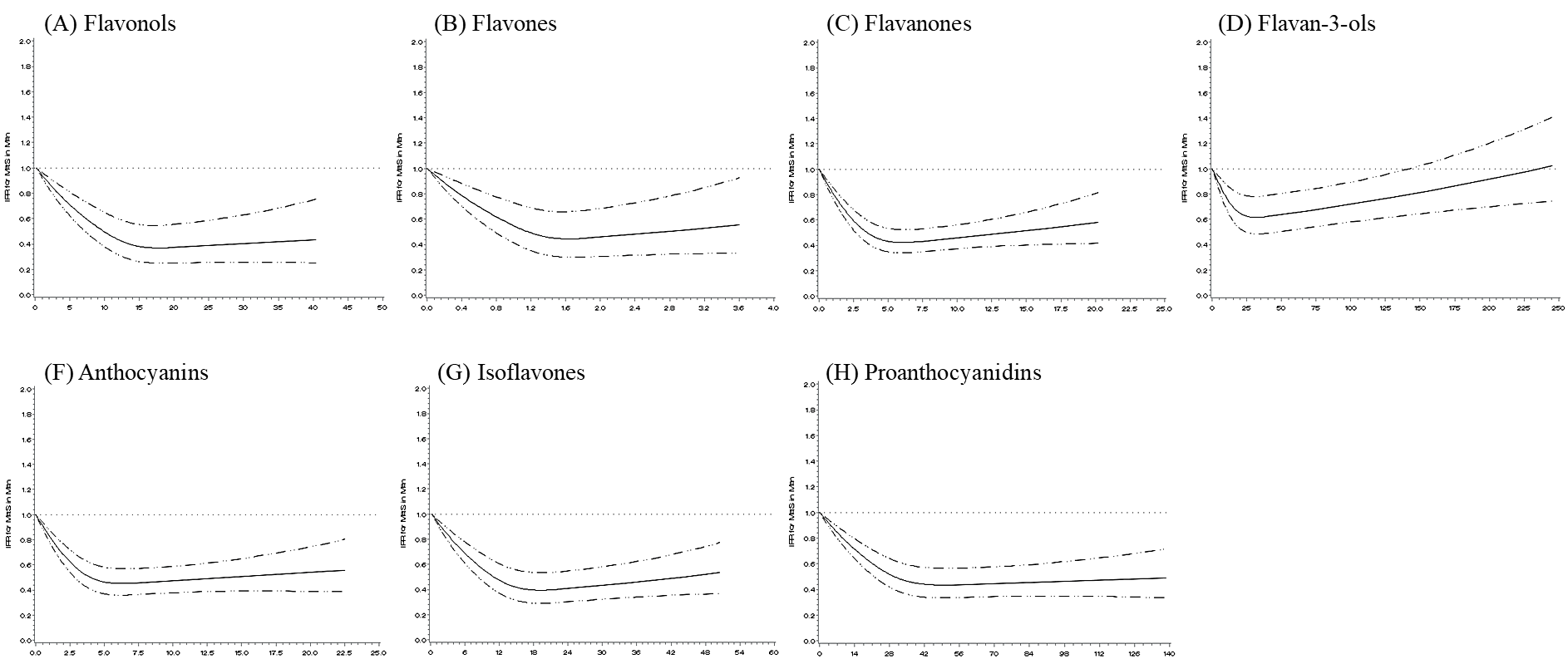


Supplemental Material 2.1. Nonlinear dose-response association of total flavonoids and flavonoid subclasses with incident MetS in men: a restricted cubic spline analysis with three knots (located at the 25th, 50th, and 75th percentiles)

MetS, metabolic syndrome; dTAC, dietary total antioxidant capacity.

Multivariable model was adjusted for age (years), higher education level (≥12 years of schooling), regular exercise (≥3 times/wk and ≥30 min/session), smoking status (current/past/never-smokers for men and current/non-smokers for women), drinking status (yes or no), body mass index (kg/m^2^), and dietary factors (total energy intake (kcal/day), glycemic index (GI), calcium (mg/day), fiber (g/day), magnesium (mg/day), and sodium (mg/day)) in men and women.


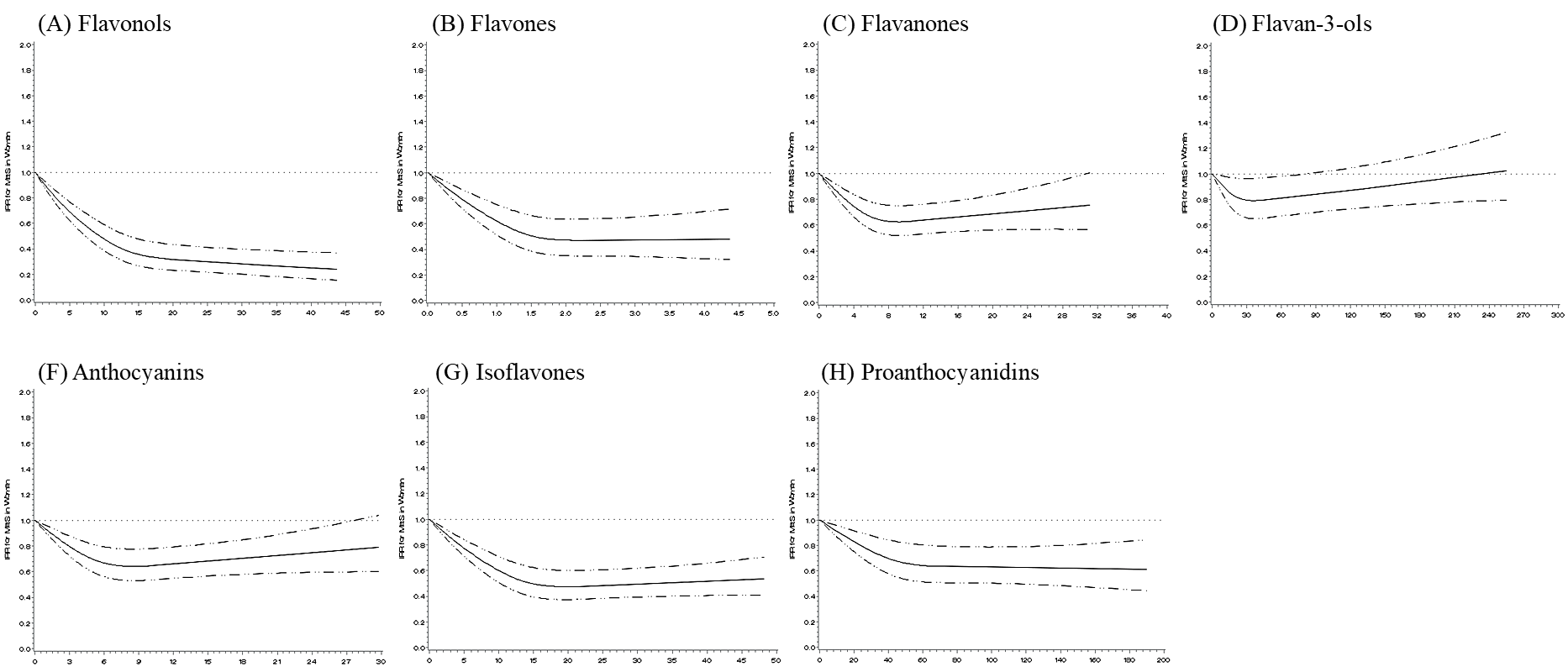


Supplemental Material 2.2. Nonlinear dose-response association of total flavonoids and flavonoid subclasses with incident MetS in women: a restricted cubic spline analysis with three knots (located at the 25th, 50th, and 75th percentiles)

MetS, metabolic syndrome; dTAC, dietary total antioxidant capacity.

Multivariable model was adjusted for age (years), higher education level (≥12 years of schooling), regular exercise (≥3 times/wk and ≥30 min/session), smoking status (current/past/never-smokers for men and current/non-smokers for women), drinking status (yes or no), body mass index (kg/m^2^), and dietary factors (total energy intake (kcal/day), glycemic index (GI), calcium (mg/day), fiber (g/day), magnesium (mg/day), and sodium (mg/day)) in men and women.
