# Supplementary material for: Associations of cumulative average dietary total antioxidant capacity and intake of antioxidants with metabolic syndrome risk in Korean adults aged 40 years and older: a prospective cohort study (KoGES_CAVAS)
Source: Epidemiol Health. 2023 Jul 28;45:e2023067. doi: 10.4178/epih.e2023067 (PMC10667584; doi:10.4178/epih.e2023067)
Supplement: Supplementary Material 3 — Baseline characteristics of the study participants (n=11,379) [file epih-45-e2023067-Supplementary-3.docx]

**Supplemental Material 3.** Baseline characteristics of the study participants (n=11,379)

| **Characteristic** | **Men (n=4,422)** | | |  | **Women (n=6,957)** | | |  | **p value**^1^ |
| --- | --- | --- | --- | --- | --- | --- | --- | --- | --- |
| Age (yr) | 59.7 ± 9.74 | | |  | 56.3 ± 9.84 | | |  | <.0001 |
| Higher education^2^ | 1,568 (35.5) | | |  | 1,930 (27.7) | | |  | <.0001 |
| Regular exercise^3^ | 921 (20.8) | | |  | 1,618 (23.3) | | |  | 0.003 |
| Smoking status |  | | |  |  | | |  |  |
| Never-smoker | 1,285 (29.1) | | |  | 6,682 (96.1) | | |  | <.0001 |
| Past smoker | 1,508 (34.1) | | |  | 79.0 (1.14) | | |  | <.0001 |
| Current smoker | 1,629 (36.8) | | |  | 196 (2.82) | | |  | <.0001 |
| Current drinker | 2,854 (64.5) | | |  | 2,085 (30.0) | | |  | <.0001 |
| Alcohol consumption (g/day) | 21.7 ± 41.7 | | |  | 1.99 ± 8.75 | | |  | <.0001 |
| Body Mass index (kg/m^2^) | 23.1 ± 2.65 | | |  | 23.5 ± 2.87 | | |  | <.0001 |
| Total energy intake (kcal/day) | 1,673 ± 439 | | |  | 1,507 ± 423 | | |  | <.0001 |
| Glycemic index | 57.3 ± 2.47 | | |  | 57.1 ± 2.97 | | |  | <.0001 |
| Calcium (mg/day) | 345 ± 137 | | |  | 348 ± 150 | | |  | 0.002 |
| Fiber (g/day) | 15.5 ± 4.63 | | |  | 14.8 ± 4.72 | | |  | 0.008 |
| Magnesium (mg/day) | 95.2 ± 18.2 | | |  | 86.7 ± 18.5 | | |  | <.0001 |
| Sodium (mg/day) | 2,869 ± 1,395 | | |  | 2,383 ± 1,256 | | |  | <.0001 |
|  |  | |  |  |  |  | |  |  |
| **Dietary antioxidant intake and capacity** | **Daily intake (mg/d)** | **Capacity**  **(mg VCE/d)** | |  | **Daily intake (mg/d)** | | **Capacity**  **(mg VCE/d)** | |  |
| Total antioxidant capacity | _ | 269 ± 279 | |  | _ | | 316 ± 343 | | <.0001 |
| Antioxidants |  |  | |  |  | |  | |  |
| *Five antioxidant classes* |  |  | |  |  | |  | |  |
| Retinol | 0.069 ± 0.068 | 0.0045 ± 0.0044 | |  | 0.069 ± 0.067 | | 0.0045 ± 0.0044 | | 0.748 |
| Vitamin C | 43.9 ± 29.8 | 43.9 ± 29.8 | |  | 52.8 ± 35.6 | | 52.8 ± 35.6 | | <.0001 |
| Vitamin E | 4.95 ± 3.15 | 1.74 ± 1.12 | |  | 4.57 ± 3.11 | | 1.61 ± 1.10 | | <.0001 |
| Carotenoids | 6.32 ± 5.34 | 2.45 ± 2.54 | |  | 7.38 ± 6.41 | | 2.94 ± 3.05 | | <.0001 |
| Flavonoids | 155 ± 143 | 221 ± 263 | |  | 187 ± 177 | | 258 ± 324 | | <.0001 |
| *Seven flavonoid subclasses* |  |  | |  |  | |  | |  |
| Flavonols | 17.6 ± 13.0 | 33.9 ± 25.6 | |  | 18.2 ± 14.9 | | 36.1 ± 29.9 | | <.0001 |
| Flavones | 1.66 ± 1.11 | 2.41 ± 1.52 | |  | 1.91 ± 1.36 | | 2.66 ± 1.82 | | <.0001 |
| Flavanones | 6.60 ± 8.82 | 8.02 ± 10.4 | |  | 9.89 ± 11.7 | | 12.0 ± 13.8 | | <.0001 |
| Flavan-3-ols | 53.1 ± 98.2 | 125 ± 232 | |  | 62.2 ± 121 | | 147 ± 285 | | <.0001 |
| Anthocyanins | 6.80 ± 8.87 | 12.0 ± 15.1 | |  | 9.48 ± 11.7 | | 16.7 ± 19.9 | | <.0001 |
| Isoflavones | 19.7 ± 16.0 | 19.4 ± 15.9 | |  | 19.1 ± 15.4 | | 18.8 ± 15.3 | | <.0001 |
| Proanthocyanidins | 49.0 ± 54.2 | 19.9 ± 19.4 | |  | 66.1 ± 67.6 | | 25.5 ± 23.4 | | <.0001 |

Values are expressed as mean ± standard deviation for continuous variables and as the number of participants (%) for categorical variables; All nutrient intakes, except components of the antioxidant capacity index, were total energy adjusted values by the residual method.

VCE, vitamin C equivalents.

^1^*p*-values were obtained by *t*-test.

^2^Higher education level (≥12 years of schooling).

^3^Regular exercise (≥3 times/wk and ≥30 min/session).
