# Supplementary material for: Associations of cumulative average dietary total antioxidant capacity and intake of antioxidants with metabolic syndrome risk in Korean adults aged 40 years and older: a prospective cohort study (KoGES_CAVAS)
Source: Epidemiol Health. 2023 Jul 28;45:e2023067. doi: 10.4178/epih.e2023067 (PMC10667584; doi:10.4178/epih.e2023067)
Supplement: Supplementary Material 4 — Incidence rate ratios (IRRs) and 95% confidence intervals (CIs) of MetS by the quartiles of dTAC and antioxidant classes after censoring CVD and cancer (n=11,379) [file epih-45-e2023067-Supplementary-4.docx]

**Supplemental Material 4.** Incidence rate ratios (IRRs) and 95% confidence intervals (CIs) of MetS by the quartiles of dTAC and intake of 5 antioxidant classes after censoring CVD and cancer (n=11,379)

|  | **Men (n=4,422)** | | | | P_linearity_^1^ | P_non-linearity_^2^ | **Women (n=6,957)** | | | | P_linearity_^1^ | P_non-linearity_^2^ |
| --- | --- | --- | --- | --- | --- | --- | --- | --- | --- | --- | --- | --- |
|  | Q1 | Q2 | Q3 | Q4 |  |  | Q1 | Q2 | Q3 | Q4 |  |  |
| **dTAC (mg VCE/day)** | 1 (ref.) | **0.71 (0.58–0.87)** | **0.78 (0.63–0.97)** | **0.77 (0.60–0.98)** | 0.4614 | **0.0029** | 1 (ref.) | 0.94 (0.81–1.10) | 0.94 (0.78–1.12) | 0.86 (0.69–1.06) | 0.1597 | 0.2910 |
| **Antioxidants (mg/day)** |  |  |  |  |  |  |  |  |  |  |  |  |
| ***Five antioxidant classes*** |  |  |  |  |  |  |  |  |  |  |  |  |
| Retinol (mg/day) | 1 (ref.) | **0.72 (0.59–0.88)** | **0.61 (0.49–0.76)** | **0.65 (0.49–0.86)** | **0.0294** | **0.0008** | 1 (ref.) | **0.75 (0.65–0.87)** | **0.64 (0.54–0.77)** | **0.73 (0.58–0.94)** | 0.0562 | **<0.0001** |
| Vitamin C (mg/day) | 1 (ref.) | **0.65 (0.54–0.79)** | **0.59 (0.47–0.74)** | **0.56 (0.41–0.75)** | **0.0026** | **0.0001** | 1 (ref.) | **0.76 (0.65–0.89)** | **0.77 (0.64–0.92)** | **0.67 (0.53–0.85)** | **0.0067** | **0.0323** |
| Vitamin E (mg/day) | 1 (ref.) | **0.70 (0.58–0.86)** | **0.59 (0.47–0.74)** | **0.71 (0.53–0.95)** | 0.1165 | **<0.0001** | 1 (ref.) | **0.79 (0.68–0.92)** | **0.66 (0.55–0.79)** | **0.75 (0.60–0.95)** | **0.0401** | **0.0026** |
| Carotenoids (mg/day) | 1 (ref.) | **0.59 (0.49–0.71)** | **0.54 (0.44–0.66)** | **0.56 (0.43–0.71)** | **0.0009** | **<0.0001** | 1 (ref.) | **0.85 (0.74–0.99)** | **0.74 (0.62–0.87)** | **0.74 (0.60–0.91)** | **0.0107** | 0.1380 |
| Flavonoids (mg/day) | 1 (ref.) | **0.63 (0.52–0.77)** | **0.69 (0.56–0.85)** | **0.66 (0.51–0.84)** | 0.0821 | **<0.0001** | 1 (ref.) | 0.90 (0.78–1.06) | 0.92 (0.77–1.10) | 0.91 (0.73–1.12) | 0.6212 | 0.2500 |
| ***Seven flavonoid subclasses*** |  |  |  |  |  |  |  |  |  |  |  |  |
| Flavonols (mg/day) | 1 (ref.) | **0.66 (0.54–0.80)** | **0.66 (0.54–0.83)** | **0.63 (0.48–0.83)** | **0.0210** | **0.0006** | 1 (ref.) | **0.82 (0.71–0.95)** | **0.62 (0.52–0.74)** | **0.64 (0.51–0.81)** | **0.0008** | **0.0010** |
| Flavones (mg/day) | 1 (ref.) | 0.84 (0.69–1.03) | 0.74 (0.60–0.93) | 0.85 (0.65–1.13) | 0.4821 | **0.0180** | 1 (ref.) | **0.79 (0.68–0.91)** | **0.67 (0.57–0.80)** | **0.73 (0.58–0.91)** | 0.0249 | **0.0160** |
| Flavanones (mg/day) | 1 (ref.) | **0.57 (0.47–0.69)** | **0.54 (0.45–0.66)** | **0.60 (0.49–0.73)** | **0.0012** | **<0.0001** | 1 (ref.) | **0.67 (0.57–0.77)** | **0.77 (0.66–0.90)** | **0.71 (0.59–0.85)** | **0.0181** | **<0.0001** |
| Flavan-3ols (mg/day) | 1 (ref.) | **0.79 (0.65–0.95)** | **0.69 (0.56–0.85)** | **0.77 (0.63–0.95)** | 0.4527 | **0.0104** | 1 (ref.) | **0.85 (0.73–0.99)** | 0.93 (0.79–1.09) | 0.84 (0.71–1.00) | 0.1688 | 0.1220 |
| Anthocyanins (mg/day) | 1 (ref.) | **0.64 (0.54–0.77)** | **0.49 (0.40–0.60)** | **0.56 (0.45–0.69)** | **0.0002** | **<0.0001** | 1 (ref.) | **0.84 (0.72–0.97)** | **0.76 (0.65–0.90)** | **0.82 (0.69–0.98)** | 0.1854 | **0.0152** |
| Isoflavones (mg/day) | 1 (ref.) | **0.76 (0.63–0.92)** | **0.57 (0.46–0.69)** | **0.72 (0.58–0.90)** | **0.0465** | **<0.0001** | 1 (ref.) | **0.76 (0.66–0.88)** | **0.63 (0.53–0.74)** | **0.72 (0.61–0.86)** | **0.0071** | **<0.0001** |
| Proanthocyanidins (mg/day) | 1 (ref.) | **0.77 (0.64–0.93)** | **0.59 (0.48–0.72)** | **0.58 (0.46–0.73)** | **<.0001** | **0.0039** | 1 (ref.) | **0.85 (0.74–0.99)** | **0.74 (0.63–0.88)** | **0.76 (0.62–0.92)** | **0.0217** | 0.0985 |

MetS, metabolic syndrome; VCE, vitamin C equivalents; dTAC, dietary total antioxidant capacity; Q, quartile.

Multivariable model was adjusted for age (years), higher education level (≥12 years), regular exercise (≥3 times/wk for ≥30 min/session), smoking (current/past/non-smokers for men and current/past and non-smokers for women), drinking status (yes or no), body mass index (kg/m^2^), total energy intake (kcal/d), glycemic index (GI), calcium (mg/d), fiber (g/d), magnesium (mg/d), and sodium (mg/d) in men and women.

^1^Linear trends were obtained by treating the median value of each group as a continuous variable.

^2^Non-linear trends were obtained by comparing the deviance difference between the linear trend model with 1 degree of freedom and the ℓ ordered categorical model with ℓ−1 degrees of freedom.
