# Supplementary material for: Associations of cumulative average dietary total antioxidant capacity and intake of antioxidants with metabolic syndrome risk in Korean adults aged 40 years and older: a prospective cohort study (KoGES_CAVAS)
Source: Epidemiol Health. 2023 Jul 28;45:e2023067. doi: 10.4178/epih.e2023067 (PMC10667584; doi:10.4178/epih.e2023067)
Supplement: Supplementary Material 5 — Incidence rate ratios (IRRs) and 95% confidence intervals (CIs) of MetS by quartiles of dTAC and antioxidant classes among only non-users of antioxidant component supplement (n=8,786) [file epih-45-e2023067-Supplementary-5.docx]

**Supplemental Material 5.** Incidence rate ratios (IRRs) and 95% confidence intervals (CIs) of MetS by quartiles of dTAC and intake of 5 antioxidant classes among only non-users of antioxidant component supplement (n=8,786)

|  | **Men (n=3,663)** | | | | P_linearity_^1^ | P_non-linearity_^2^ | **Women (n=5,123)** | | | | P_linearity_^1^ | P_non-linearity_^2^ |
| --- | --- | --- | --- | --- | --- | --- | --- | --- | --- | --- | --- | --- |
|  | Q1 | Q2 | Q3 | Q4 |  |  | Q1 | Q2 | Q3 | Q4 |  |  |
| **dTAC (mg VCE/day)** | 1 (ref.) | **0.73 (0.59–0.89)** | **0.74 (0.59–0.93)** | 0.78 (0.60–1.01) | 0.5653 | **0.0062** | 1 (ref.) | 0.91 (0.77–1.08) | 1.01 (0.83–1.23) | 0.91 (0.72–1.15) | 0.5599 | 0.3350 |
| **Antioxidants (mg/day)** |  |  |  |  |  |  |  |  |  |  |  |  |
| ***Five antioxidant classes*** |  |  |  |  |  |  |  |  |  |  |  |  |
| Retinol (mg/day) | 1 (ref.) | **0.68 (0.55–0.83)** | **0.62 (0.49–0.77)** | **0.61 (0.46–0.82)** | **0.0180** | **0.0005** | 1 (ref.) | **0.78 (0.67–0.91)** | **0.65 (0.54–0.79)** | **0.73 (0.56–0.96)** | 0.0446 | **0.0003** |
| Vitamin C (mg/day) | 1 (ref.) | **0.61 (0.50–0.75)** | **0.58 (0.46–0.73)** | **0.57 (0.42–0.78)** | **0.0072** | **<0.0001** | 1 (ref.) | **0.80 (0.68–0.94)** | **0.80 (0.65–0.98)** | **0.76 (0.58–1.00)** | 0.1260 | 0.0718 |
| Vitamin E (mg/day) | 1 (ref.) | **0.70 (0.57–0.86)** | **0.61 (0.48–0.77)** | **0.73 (0.54–0.99)** | 0.1446 | **<0.0001** | 1 (ref.) | **0.84 (0.71–0.99)** | **0.70 (0.58–0.86)** | **0.76 (0.59–0.98)** | 0.0395 | **0.0161** |
| Carotenoids (mg/day) | 1 (ref.) | **0.59 (0.48–0.71)** | **0.58 (0.47–0.72)** | **0.54 (0.42–0.71)** | **0.0009** | **<0.0001** | 1 (ref.) | 0.90 (0.77–1.06) | **0.77 (0.63–0.93)** | 0.80 (0.63–1.01) | 0.0640 | 0.1170 |
| Flavonoids (mg/day) | 1 (ref.) | **0.61 (0.50–0.74)** | **0.67 (0.54–0.84)** | **0.65 (0.50–0.85)** | 0.1263 | **<0.0001** | 1 (ref.) | 0.92 (0.78–1.09) | 1.00 (0.82–1.20) | 0.95 (0.75–1.20) | 0.8859 | 0.5170 |
| ***Seven flavonoid subclasses*** |  |  |  |  |  |  |  |  |  |  |  |  |
| Flavonols (mg/day) | 1 (ref.) | **0.62 (0.50–0.75)** | **0.67 (0.53–0.83)** | **0.62 (0.46–0.82)** | **0.0261** | **0.0001** | 1 (ref.) | **0.81 (0.69–0.95)** | **0.62 (0.51–0.75)** | **0.68 (0.52–0.88)** | **0.0104** | **0.0003** |
| Flavones (mg/day) | 1 (ref.) | **0.79 (0.64–0.97)** | **0.74 (0.58–0.93)** | 0.87 (0.66–1.17) | 0.7008 | **0.0075** | 1 (ref.) | **0.80 (0.68–0.95)** | **0.73 (0.60–0.88)** | 0.79 (0.62–1.01) | 0.1425 | **0.0080** |
| Flavanones (mg/day) | 1 (ref.) | **0.61 (0.50–0.74)** | **0.54 (0.45–0.66)** | **0.65 (0.53–0.81)** | **0.0075** | **<0.0001** | 1 (ref.) | **0.68 (0.58–0.80)** | **0.78 (0.66–0.93)** | **0.73 (0.6–0.89)** | **0.0456** | **<0.0001** |
| Flavan–3ols (mg/day) | 1 (ref.) | 0.87 (0.72–1.06) | **0.71 (0.57–0.88)** | **0.78 (0.63–0.98)** | 0.2573 | **0.0111** | 1 (ref.) | 0.89 (0.76–1.05) | 1.02 (0.85–1.21) | 0.90 (0.74–1.09) | 0.4143 | 0.1910 |
| Anthocyanins (mg/day) | 1 (ref.) | **0.69 (0.57–0.83)** | **0.54 (0.44–0.66)** | **0.61 (0.49–0.76)** | **0.0018** | **<0.0001** | 1 (ref.) | **0.84 (0.72–0.98)** | **0.79 (0.66–0.94)** | 0.85 (0.70–1.04) | 0.3577 | **0.0260** |
| Isoflavones (mg/day) | 1 (ref.) | **0.80 (0.66–0.96)** | **0.56 (0.45–0.69)** | **0.72 (0.57–0.90)** | **0.0264** | **<0.0001** | 1 (ref.) | **0.80 (0.68–0.93)** | **0.61 (0.51–0.73)** | **0.77 (0.64–0.93)** | **0.0374** | **<0.0001** |
| Proanthocyanidins (mg/day) | 1 (ref.) | **0.74 (0.61–0.89)** | **0.57 (0.46–0.70)** | **0.58 (0.46–0.74)** | **<.0001** | **0.0008** | 1 (ref.) | 0.87 (0.75–1.02) | **0.79 (0.66–0.95)** | **0.78 (0.62–0.97)** | **0.0496** | 0.1830 |

MetS, metabolic syndrome; VCE, vitamin C equivalents; dTAC, dietary total antioxidant capacity; Q, quartile.

Multivariable model was adjusted for age (years), higher education level (≥12 years), regular exercise (≥3 times/wk for ≥30 min/session), smoking (current/past/non-smokers for men and current/past and non-smokers for women), drinking status (yes or no), body mass index (kg/m^2^), total energy intake (kcal/d), glycemic index (GI), calcium (mg/d), fiber (g/d), magnesium (mg/d), and sodium (mg/d) in men and women.

^1^Linear trends were obtained by treating the median value of each group as a continuous variable.

^2^Non-linear trends were obtained by comparing the deviance difference between the linear trend model with 1 degree of freedom and the ℓ ordered categorical model with ℓ−1 degrees of freedom.
