# Supplementary material for: Associations of cumulative average dietary total antioxidant capacity and intake of antioxidants with metabolic syndrome risk in Korean adults aged 40 years and older: a prospective cohort study (KoGES_CAVAS)
Source: Epidemiol Health. 2023 Jul 28;45:e2023067. doi: 10.4178/epih.e2023067 (PMC10667584; doi:10.4178/epih.e2023067)
Supplement: Supplementary Material 6 — Incidence rate ratios (IRRs) and 95% confidence intervals (CIs) of MetS by quartiles of dTAC and antioxidant classes of their major food sources (n=11,379) [file epih-45-e2023067-Supplementary-6.docx]

**Supplemental Material 6.** Incidence rate ratios (IRRs) and 95% confidence intervals (CIs) of MetS by quartiles of dTAC and antioxidant classes of their major food sources (n=11,379)

|  | **Food item** | **Men (n=4,422)** | | | | |  | **Women (n=6,957)** | | | | |
| --- | --- | --- | --- | --- | --- | --- | --- | --- | --- | --- | --- | --- |
|  |  | Contribution to intake (%)^1^ | Contribution to Variation (%)^2^ | Quartiles of antioxidants | | |  | Contribution to intake (%)^1^ | Contribution to Variation (%)^2^ | Quartiles of antioxidants | | |
|  |  |  |  | Q2 | Q3 | Q4 |  |  |  | Q2 | Q3 | Q4 |
| **dTAC (mgVCE/day)** | Green tea | 23.2 | 87.3 | 0.84 (0.68–1.04) | 0.76 (0.64–0.90) | 0.77 (0.65–0.91) |  | 22.8 | 87.6 | 0.77 (0.66–0.91) | 0.83 (0.73–0.95) | 0.78 (0.68–0.90) |
| **Antioxidants (mg/day)** | |  |  |  |  |  |  |  |  |  |  |  |
| ***Five antioxidant classes*** | |  |  |  |  |  |  |  |  |  |  |  |
| Retinol | Milk | 27.5 | 65.0 | 0.56 (0.46–0.68) | 0.68 (0.57–0.80) | 0.66 (0.53–0.81) |  | 34.9 | 73.2 | 0.69 (0.60–0.80) | 0.56 (0.49–0.65) | 0.53 (0.44–0.64) |
|  | Egg/ quail egg | 26.2 | 13.1 | 0.57 (0.48–0.68) | 0.58 (0.49–0.70) | 0.50 (0.42–0.61) |  | 23.3 | – | 0.83 (0.73–0.94) | 0.69 (0.60–0.80) | 0.52 (0.44–0.61) |
| Vitamin C | *Kimchi, Baechu-kimchi* | 16.6 | – | 0.88 (0.73–1.06) | 0.37 (0.28–0.48) | 1.43 (1.14–1.78) |  | 21.4 | – | 1.13 (0.98–1.30) | 0.46 (0.39–0.55) | 1.39 (1.16–1.67) |
|  | Tangerine | 12.2 | 10.5 | 0.76 (0.64–0.89) | 0.40 (0.33–0.49) | 0.72 (0.61–0.86) |  | – | 33.8 | 0.64 (0.56–0.74) | 0.63 (0.55–0.73) | 0.82 (0.70–0.96) |
|  | Strawberries | – | 33.3 | 0.62 (0.52–0.74) | 0.60 (0.50–0.72) | 0.78 (0.64–0.95) |  | – | 12.0 | 0.73 (0.63–0.85) | 0.79 (0.69–0.92) | 0.82 (0.70–0.96) |
|  | Orange/orange juice | – | 21.8 | 0.22 (0.13–0.39) | 0.57 (0.48–0.67) | 0.77 (0.66–0.92) |  | – | 20.3 | 0.58 (0.49–0.70) | 0.59 (0.52–0.68) | 0.78 (0.68–0.90) |
| Vitamin E | Tofu | 18.5 | 34.7 | 0.80 (0.67–0.95) | 0.88 (0.74–1.05) | 0.71 (0.58–0.86) |  | 17.4 | 38.5 | 0.70 (0.60–0.80) | 0.89 (0.77–1.03) | 0.76 (0.65–0.90) |
|  | Soy milk | – | 19.0 | 0.58 (0.49–0.70) | 0.59 (0.51–0.68) |  |  | – | 18.1 | 0.39 (0.31–0.49) | 0.74 (0.66–0.83) |  |
| Carotenoids | Watermelon | 17.2 | – | 0.61 (0.52–0.72) | 0.55 (0.46–0.66) | 0.48 (0.40–0.57) |  | 15.7 | 10.0 | 0.76 (0.66–0.87) | 0.80 (0.69–0.93) | 0.98 (0.85–1.14) |
|  | Tomato/tomato juice/cherry tomato | 26.8 | 71.3 | 0.50 (0.41–0.60) | 0.66 (0.55–0.78) | 0.58 (0.48–0.70) |  | 24.2 | 71.9 | 0.77 (0.68–0.89) | 0.50 (0.43–0.58) | 0.70 (0.60–0.82) |
|  | Spinach | – | 11.5 | 0.55 (0.46–0.66) | 0.63 (0.53–0.74) | 0.58 (0.48–0.70) |  | – | – |  |  |  |
| Flavonoids | Green tea | 18.4 | 67.0 | 0.84 (0.68–1.04) | 0.76 (0.65–0.90) | 0.77 (0.65–0.91) |  | 19.2 | 67.4 | 0.77 (0.66–0.91) | 0.83 (0.73–0.95) | 0.78 (0.68–0.90) |
|  | Apple/apple juice | 14.3 | 19.5 | 0.50 (0.42–0.59) | 0.54 (0.46–0.64) | 0.42 (0.34–0.50) |  | 11.6 | 19.8 | 0.83 (0.73–0.95) | 0.61 (0.52–0.70) | 0.68 (0.58–0.80) |
|  | Grapes/grapes juice | 12.7 | – | 0.52 (0.43–0.62) | 0.54 (0.45–0.65) | 0.62 (0.52–0.75) |  | 10.8 | – | 0.78 (0.68–0.90) | 0.63 (0.54–0.73) | 0.61 (0.52–0.72) |
| ***Seven flavonoid subclasses*** | |  |  |  |  |  |  |  |  |  |  |  |
| Flavonols | Lettuce | 17.9 | 16.8 | 0.89 (0.75–1.06) | 0.63 (0.52–0.77) | 0.96 (0.80–1.16) |  | 17.4 | 20.4 | 0.90 (0.79–1.03) | 0.48 (0.41–0.56) | 0.76 (0.66–0.89) |
|  | Other green vegetables | 12.2 | 45.7 | 0.44 (0.37–0.52) | 0.45 (0.38–0.53) | 0.56 (0.47–0.67) |  | – | 47.0 | 0.77 (0.68–0.88) | 0.59 (0.51–0.68) | 0.54 (0.46–0.63) |
|  | Green tea | – | 10.2 | 0.84 (0.68–1.04) | 0.76 (0.65–0.90) | 0.77 (0.65–0.91) |  | – | 12.4 | 0.77 (0.66–0.91) | 0.83 (0.73–0.95) | 0.78 (0.68–0.90) |
|  | Radish Kimchi (*Kkakdugi* etc.) | – | – |  |  |  |  | 11.6 | – | 0.66 (0.58–0.76) | 0.63 (0.55–0.73) | 0.58 (0.49–0.69) |
|  | Other Kimchi (*Pakimchi/Kodulbbagi/Gatkimchi*) | – | 13.5 | 0.41 (0.34–0.50) | 0.64 (0.54–0.75) | 0.50 (0.42–0.59) |  | – | – |  |  |  |
| Flavones | Green pepper | 18.8 | 40.2 | 0.73 (0.61–0.88) | 0.74 (0.62–0.89) | 0.86 (0.71–1.04) |  | 19.0 | 38.4 | 0.89 (0.78–1.02) | 0.49 (0.41–0.58) | 0.77 (0.65–0.90) |
|  | Tangerine | 17.9 | 12.2 | 0.76 (0.64–0.89) | 0.40 (0.33–0.49) | 0.72 (0.61–0.86) |  | 13.7 | 24.9 | 0.64 (0.56–0.74) | 0.63 (0.55–0.73) | 0.82 (0.70–0.96) |
|  | *Kimchi, Baechu-kimchi* | 14.1 | – | 0.95 (0.79–1.15) | 0.33 (0.25–0.42) | 1.29 (1.03–1.61) |  | 17.2 | – | 1.02 (0.89–1.18) | 0.44 (0.37–0.52) | 1.28 (1.06–1.54) |
|  | Orange/orange juice | – | 23.0 | 0.21 (0.12–0.37) | 0.58 (0.49–0.68) | 0.78 (0.66–0.92) |  | – | 15.9 | 0.58 (0.49–0.70) | 0.59 (0.52–0.68) | 0.78 (0.68–0.90) |
| Flavanones | Tangerine | 51.2 | 28.0 | 0.76 (0.64–0.89) | 0.40 (0.33–0.49) | 0.72 (0.61–0.86) |  | 49.0 | 33.3 | 0.64 (0.56–0.74) | 0.63 (0.55–0.73) | 0.82 (0.70–0.96) |
|  | Grapes/grapes juice | 25.3 | – | 0.52 (0.43–0.62) | 0.54 (0.45–0.65) | 0.62 (0.52–0.75) |  | 26.7 | – | 0.78 (0.68–0.90) | 0.63 (0.54–0.73) | 0.61 (0.52–0.72) |
|  | Orange/orange juice | 19.2 | 62.1 | 0.22 (0.13–0.39) | 0.57 (0.48–0.67) | 0.77 (0.66–0.92) |  | 18.0 | 57.2 | 0.58 (0.49–0.70) | 0.59 (0.52–0.68) | 0.78 (0.68–0.90) |
| Flavan-3-ols | Green tea | 45.4 | 99.5 | 0.84 (0.68–1.04) | 0.76 (0.65–0.90) | 0.77 (0.65–0.91) |  | 45.3 | 99.5 | 0.77 (0.66–0.91) | 0.83 (0.73–0.95) | 0.78 (0.68–0.90) |
|  | Grapes/grapes juice | 13.5 | – | 0.52 (0.43–0.62) | 0.54 (0.45–0.65) | 0.62 (0.52–0.75) |  | 11.7 | – | 0.78 (0.68–0.90) | 0.63 (0.54–0.73) | 0.61 (0.52–0.72) |
| Anthocyanins | Grapes/grapes juice | 50.4 | 87.1 | 0.52 (0.43–0.62) | 0.54 (0.45–0.65) | 0.62 (0.52–0.75) |  | 47.2 | 88.3 | 0.78 (0.68–0.90) | 0.63 (0.54–0.73) | 0.61 (0.52–0.72) |
|  | Strawberries | 24.4 | 10.7 | 0.62 (0.52–0.74) | 0.60 (0.50–0.72) | 0.78 (0.64–0.95) |  | 25.3 | – | 0.74 (0.64–0.85) | 0.80 (0.69–0.92) | 0.83 (0.70–0.97) |
|  | Apple/apple juice | 10.0 | – | 0.49 (0.41–0.59) | 0.54 (0.45–0.63) | 0.41 (0.34–0.50) |  | – | – | 0.84 (0.73–0.96) | 0.61 (0.53–0.71) | 0.68 (0.58–0.81) |
| Isoflavones | Tofu | 26.0 | 28.0 | 0.80 (0.67–0.95) | 0.88 (0.73–1.05) | 0.71 (0.58–0.87) |  | 26.0 | 30.0 | 0.69 (0.60–0.80) | 0.89 (0.77–1.02) | 0.77 (0.65–0.91) |
|  | Fermented soybean products^3^ | 19.1 | – | 0.92 (0.77–1.10) | 0.90 (0.75–1.08) | 0.92 (0.76–1.11) |  | 19.2 | – | 0.97 (0.84–1.12) | 0.88 (0.76–1.01) | 0.90 (0.77–1.04) |
|  | Cooked rice with multi-grains | 12.5 | – | 0.38 (0.31–0.46) | 0.70 (0.60–0.82) |  |  | – | – |  |  |  |
|  | Cooked rice with beans | 10.3 | 39.1 | 0.69 (0.58–0.84) |  |  |  | 10.1 | 33.9 | 0.65 (0.57–0.75) |  |  |
|  | Soybeans/soybeans cooked in soy sauce | – | 17.1 | 0.30 (0.22–0.40) | 0.60 (0.51–0.71) | 0.71 (0.60–0.83) |  | – | 20.0 | 0.27 (0.21–0.35) | 0.66 (0.58–0.75) | 0.61 (0.53–0.70) |
| Proanthocyanidins | Apple/apple juice | 30.1 | 78.5 | 0.50 (0.42–0.59) | 0.54 (0.46–0.64) | 0.42 (0.34–0.50) |  | 26.9 | 79.1 | 0.84 (0.73–0.96) | 0.61 (0.53–0.71) | 0.68 (0.58–0.81) |
|  | Grapes/grapes juice | 18.0 | 12.5 | 0.52 (0.43–0.62) | 0.54 (0.45–0.65) | 0.62 (0.52–0.75) |  | 16.7 | 13.7 | 0.79 (0.69–0.91) | 0.62 (0.54–0.72) | 0.61 (0.52–0.71) |
|  | Cooked rice with multi-grains | 13.6 | – | 0.39 (0.32–0.48) | 0.68 (0.58–0.80) |  |  | 13.0 | – | 0.36 (0.31–0.41) | 0.73 (0.64–0.82) |  |
|  | Strawberries | 13.1 | – | 0.62 (0.52–0.74) | 0.60 (0.50–0.72) | 0.78 (0.64–0.95) |  | 13.1 | – | 0.73 (0.63–0.85) | 0.79 (0.69–0.92) | 0.82 (0.70–0.96) |

MetS, metabolic syndrome; VCE, vitamin C equivalents; dTAC, dietary total antioxidant capacity; Q, quartile.

IRRs were obtained by multivariable model after adjusting for age (years), higher education level (≥12 years), regular exercise (≥3 times/wk for ≥30 min/session), smoking (current/pass/non-smokers for men and current/non-smokers for women), drinking status (yes or no), body mass index (kg/m^2^), and total energy intake (kcal/day), glycemic index (GI), calcium (mg/day), fiber (g/day), magnesium (mg/day), and sodium (mg/day) in men and women.

^1^Only food items contributed ≥10% of either intake or variation of dTAC and antioxidants are shown.

^2^Variation (%) was partial r^2^ obtained from the multiple stepwise regression using dTAC or each antioxidant as dependent variable and antioxidant capacity or each antioxidant intake from each food item as independent variables.

^3^Fermented soybean products includes *doenjang, chengguk-jang, and ssamjang*.
